# *Astragalus mongholicus* Bunge and *Curcuma aromatica* Salisb. inhibits liver metastasis of colon cancer by regulating EMT via the CXCL8/CXCR2 axis and PI3K/AKT/mTOR signaling pathway

**DOI:** 10.1186/s13020-022-00641-4

**Published:** 2022-08-03

**Authors:** Fuyan Liu, Yan Liang, Ruolan Sun, Weicheng Yang, Zhongqing Liang, Junfei Gu, Fan Zhao, Decai Tang

**Affiliations:** 1 School of Traditional Chinese Medicine and School of Integrated Chinese and Western Medicine, Nanjing University of Chinese Medicine, Nanjing, China; 2 School of Chinese Materia Medica, Nanjing University of Chinese Medicine, Nanjing, China

**Keywords:** Liver metastasis of Colon cancer, Traditional Chinese Medicine, CXCL8, PI3K/AKT signaling pathway, EMT

## Abstract

**Background:**

One of the most challenging aspects of colon cancer (CC) prognosis and treatment is liver-tropic metastasis. *Astragalus mongholicus* Bunge—*Curcuma aromatica* Salisb. (AC) is a typical medication combination for the therapy of many malignancies. Our previous studies found that AC intervention inhibits liver metastasis of colon cancer (LMCC). Nevertheless, the comprehensive anti-metastasis mechanisms of AC have not been uncovered.

**Methods:**

In bioinformatics analysis, RNA-seq data of CC and LMCC patients were collected from TCGA and GEO databases, and differentially expressed genes (DEGs) were identified. The biological processes and signaling pathways involved in DEGs were enriched by GO and KEGG. The protein–protein interaction (PPI) network of DEGs was established and visualized using the Cytocape software, followed by screening Hub genes in the PPI network using Degree value as the criterion. Subsequently, the expression and survival relevance of Hub gene in COAD patients were verified. In the experimental study, the effects of AC on the inhibition of colon cancer growth and liver metastasis were comprehensively evaluated by cellular and animal models. Finally, based on the results of bioinformatics analysis, the possible mechanisms of AC inhibition of colon cancer EMT and liver metastasis were explored by in vivo and in vitro pharmacological experiments.

**Results:**

In this study, we obtained 2386 DEGs relevant to LMCC from the COAD (colon adenocarcinoma) and GSE38174 datasets. Results of GO gene function and KEGG signaling pathway enrichment analysis suggested that cellular EMT (Epithelial-mesenchymal transition) biological processes, Cytokine-cytokine receptor interaction and PI3K/Akt signaling pathways might be closely related to LMCC mechanism. We then screened for CXCL8, the core hub gene with the highest centrality within the PPI network of DEGs, and discovered that CXCL8 expression was negatively correlated with the prognosis of COAD patients. In vitro and in vivo experimental evidence presented that AC significantly inhibited colon cancer cell proliferation, migration and invasion ability, and suppressed tumor growth and liver metastasis in colon cancer orthotopic transplantation mice models. Concomitantly, AC significantly reduced CXCL8 expression levels in cell supernatants and serum. Moreover, AC reduced the expression and transcription of genes related to the PI3K/AKT pathway while suppressing the EMT process in colon cancer cells and model mice.

**Conclusions:**

In summary, our research predicted the potential targets and pathways of LMCC, and experimentally demonstrated that AC might inhibit the growth and liver metastasis in colon cancer by regulating EMT via the CXCL8/CXCR2 axis and PI3K/AKT/mTOR signaling pathway, which may facilitate the discovery of mechanisms and new therapeutic strategies for LMCC.

**Supplementary Information:**

The online version contains supplementary material available at 10.1186/s13020-022-00641-4.

## Background

Colorectal cancer (CRC), incorporating both colon and rectal cancers, is one of the highest prevalent gastrointestinal malignancies worldwide [1, 2], while colon cancer (CC) has a greater illness incidence than rectal cancer (RC) [3]. Nearly 90% of CRC-related deaths are triggered by distant invasion and metastasis, and the hepatic is the primary location for CRC metastasis [4]. Therefore, liver metastasis is a major challenge for the prognosis and therapy of CRC. Noteworthy, colon cancer patients are more likely to develop liver metastases than rectal cancer patients, which may results from the different directions of hematogenous metastasis [5]. Thus, CC patients with liver metastasis are a separate category that merits additional investigation and is selected for the present study.

Traditional Chinese Medicine (TCM) could suppress the tumor cells proliferation, migration and invasion, induce cellular autophagy, stimulate apoptosis, block angiogenesis and improve the immunity of the body by altering the tumor microenvironment, thus acting as a comprehensive anti-tumor development and metastasis [6, 7]. Several researches indicated that TCMs can be an effective supplemental strategy to adjuvant chemotherapy in the clinical treatment of CRC patients, and prolonged TCM therapy time can better improve the prognosis and survival rate of CRC patients [8, 9]. The traditional medication formula of *Astragalus mongholicus* Bunge and *Curcuma aromatica* Salisb. (AC), initially documented in "Yixuezhongzhongcanxilu", and statistical analysis reported that AC have significant clinical effects on inflammatory diseases and malignant tumors of the gastrointestinal system [10]. In our previous studies, we found favorable effectiveness of AC in the treatment of ovarian, hepatic and colon malignancies, especially for the prevention of liver metastasis in colon cancer orthotopic transplantation mice models [11–13]. Nonetheless, the exact mechanism of action of AC in colon cancer development and liver metastasis remains unknown.

Enormous number of CC-related microarray data has been generated, uploaded, and stored in public databases as a result of the development of high-throughput sequencing technology and genomics, as well as the accumulation of clinical data on tumor patients [14, 15]. Nevertheless, in independent investigations or single cohort studies, the results are usually restricted or inconsistent due to tissue or sample heterogeneity. As a result, no reliable biomarkers for CC have been discovered [16, 17]. However, an integrated strategy combining microarray data with bioinformatics knowledge would be innovative and may potentially overcome these flaws [18, 19]. The Cancer Genome Atlas (TCGA) and Gene Expression Omnibus (GEO) databases are well-known free resources for finding novel biomarkers in cancer research [20–22]. Reanalysis and integration of these datasets may yield valuable insights for future research.

In this work, we utilized bioinformatics analysis to predict the possible targets and pathways for the treatment of LMCC. Then, using colon cancer cell lines and orthotopic transplantation animal models, we evaluated the anticancer effects and possible mechanism of AC in vitro and in vivo. As a result, these results might open up a new perspective for study into the mechanism of colon cancer liver metastasis, as well as clinical medication development.

## Methods

### Materials

Thermo Fisher Scientific (Waltham, MA) delivered all the DMEM and other cultural medium components. 5-Fluorouracil was bought from Sigma-Aldrich (St. Louis, MO). RNAiso plus kit, PrimeScript™ RT Master Mix, TB Green® Premix Ex Taq™ II were all obtained from Vazyme (Nanjing, China). Antibodies for PI3K (#4292S), p-PI3K (Tyr458, #4228S), AKT (#9272S), p-AKT (Ser473, #9271S), E-Cadherin (#3195S), N-Cadherin (#4061S), Vimentin (#3932S), Snail (#3879S) and GAPDH (#2118S) were acquired from Cell Signaling Technology (Danvers, MA, USA). CXCR2 (ab65968) and CXCR1 (NBP2-16043) antibodies were obtained from Abcam (Cambridge, UK) and Novus Biologicals (USA).

### Bioinformatic prediction analyses

#### Data collection

We downloaded TCGA RNA-seq transcriptome data for tumor and adjacent normal tissues from colon cancer patients (COAD) from the UCSC Xena browser (https://xenabrowser.net/datapages/). We downloaded RNA-seq data of normal colonic mucosa and liver metastasis tissues from colon cancer from the GEO (https://www.ncbi.nlm.nih.gov/geo/).

#### Identification of DEGs

The COAD RNA-seq dataset were normalized for expression values adopting the edgeR package in R language software (version R × 64 4.1.0), and the limma package was used to screen out differentially expressed genes (DEGs). Use the GEO2R plugin based on the "limma" R package to screen DEGs in the GEO dataset RNA-seq. Screening criteria: false-discovery rate (FDR) < 0.05, **|**log2 fold-change**|**≥ 2.

#### Gene set enrichment analyses

GO functional enrichment analyses and KEGG pathway enrichment were conducted on differentially expressed genes adopting the DAVID database (https://david.abcc.ncifcrf.gov/).

#### PPI network analysis and visualization

A protein–protein interaction (PPI) network was built through the combination of the Cytoscape software (version 3.7.2; https://cytoscape.org/) and STRING website (version 11.0, https://cn.string-db.org/) to clarify the underlying molecular mechanisms of LMCC. Only the interactions with confidence score > 0.4 were considered as signification. Then, CytoHubba and MCODE plug-ins were used to analyze the degree of connectivity of DEGs in the PPI network and screen out the core functional modules and top 10 hub genes.

#### Expression and survival analysis of hub genes in COAD

The expression levels of hub genes in COAD patients were performed by GEPIA2 (Gene Expression Profiling Interactive Analysis 2) website (http://gepia2.cancer-pku.cn/), an online tool for cancer and normal gene expression analysis based on TCGA and GTEx (Genotype -Tissue Expression) dataset. The results are presented by box plots [23]. Furthermore, to screen out the survival-associated genes, Kaplan–Meier survival analysis was using the Oncolnc platform (https://www.oncolnc.org/). At last, GraphPad Prism software (San Diego, United States) was utilized to analyze ROC curve.

### Preparation of AC extract

The dried roots of *Astragalus mongholicus* Bunge [Fabaceae] was donated by the Jiangsu Provincial Hospital of Traditional Chinese Medicine. (Nanjing, China) and dried rhizomes of *Curcuma aromatica* Salisb. [Zingiberaceae] supplied by Tongling Hetian Traditional Chinese Medicine Decoction Pieces Co., Ltd. (Tongling, China). The pieces were identified by Professor Tulin Lu of Nanjing University of Chinese Medicine. Initially, *Astragalus mongholicus* Bunge (100 g) and *Curcuma aromatica* Salisb. (50 g) (weight ratio 2:1) were soaked in a round-bottomed flask with 1.5L of distilled water for 60 min, then decocted twice for one hour each time by the heat-reflux system. Meanwhile, the Soxhlet extractor was used to recover the essential volatile oil components. Then, the two times extracts were mixed with volatile oil and freeze-dried to powder. Finally, the extraction rate of the herb was calculated as 13.81%. The freeze-dried extract powder samples are deposited in the Key Laboratory of High Technology of Prescription Research in Jiangsu Province, Nanjing, China (voucher specimen: NO. 20190654-AC).

In this work, the experimental doses for rats and mice were calculated based on a conversion to the ratio of body surface area to human. According to the results from our earlier experimental researches, the clinical doses of *Astragalus mongholicus* Bunge 30 g and *Curcuma aromatica* Salisb. 15 g were selected [13, 24]. The doses administered of rats and mice were converted as follows: Rats: (30 + 15) g/day [Human dosage]*0.018[Rats and human body surface area ratio]/200 g[Body weight of rats] = 4.05 g/kg(≈4 g/kg); Mice: (30 + 15) g/day [Human dosage] *0.0026[Mice and human body surface area ratio]/20 g [Body weight of mice] = 5.85 g/kg(≈6 g/kg).

### Preparation of AC-containing serum

Approximately 200 g male Sprague–Dawley (SD) rats were supplied from Hangzhou Medical College, Hangzhou, China (license number: SCXK (Zhe) 2019–0002), and raised in a specific-pathogen-free environment in the Animal Experimental Center at Nanjing University of Chinese Medicine. All experimental procedures were in agreement with the Ethics Committee of the Nanjing University of Chinese Medicine, Nanjing, China (Ethics Committee approval No. 202105A045). A total of twenty rats were assigned arbitrarily to the blank control or the AC treatment group, ten rats in each group. The AC treatment group received an intragastric injection of AC (4 g/kg/day) for seven days, whereas the blank control group received the equivalent physiological saline. One hour after the final dose, rats were anesthetized with 2% isoflurane, and blood was obtained through the abdominal aorta and centrifuged at 3000 rpm for 15 min to remove plasma to yield serum. All serum specimens were inactivated by heating at 56 °C for 30 min to completely annihilate the complement activity.

The quality control methods for AC extracts and AC-containing serum were referenced to our previous study [25], and the results are detailed in Additional file 1: Tables S1–S2 and Figures S1–S3.

### *Cell-Based *In vitro* pharmacological assays*

#### Cell culture

CT26.WT cell line (BALB/c colon origin) was acquired from the Beijing Cancer Research Institute. Mouse normal hepatocyte line NCTC1469 was provided by Nanjing Kexin Bio-Tech Co. LTD. All the cells were maintained in DMEM medium containing 10% heat-inactivated fetal bovine serum, 100U/mL penicillin, and 100 μg/mL streptomycin under 37 °C, 5% CO_2_ (Corning, USA).

#### Cell viability assay

Cell proliferation was monitored with CCK8 assay kit (Ford Biological, China) as directed by the manufacturer. To be specific, CT26.WT at 8 × 10^3^ cells/well were coated onto 96-well plates overnight. Subsequently, AC serum and blank control rat serum at 5%, 10%, 15%, and 20% concentrations were added. For 24 h or 48 h of treatment, 10μL of CCK8 solution was applied to each well and incubated for one more hour, and the optical density values (OD) of the groups were then determined at 450 nm via a Multiscan Spectrum (Multiscan FC, USA). The inhibition rate (%) was calculated adopting: [(1-OD of treated group/OD of the model control group) × 100%].

#### Wound healing assay

In the collective migration experiments for scratch healing, we explored the influence of the AC serum on the migratory capacity of CT26.WT cells. Briefly, the CT26.WT cells (1 × 10^5^ cells/well) were cultured into 6-well plates supplemented growth medium and grown to approximately 90% density. Following incubation, the monolayers of CT26.WT cells were lightly scratched with a 100 μL sterile pipette tip to form several mutually perpendicular scratches, subsequently washing with PBS to rinse dislodged cells. Then the different concentration gradients of AC-containing serum were added. After 48 h, the wound gap area was monitored, and the representative pictures were snapped at 0 h, 24 h, and 48 h, respectively, utilizing a microscope matched with a digital camera (magnification,×200). Analysis of the average distance of cell migration adopting ImageJ software.

#### Transwell invasion assay

Next, to evaluate cell invasion, 2 × 10^5^ cells/well CT26.WT cells were suspended with a10% serum-free medium with or without AC-containing serum, subsequently cultured into the upper chambers of 24-well plates (8 μm, Costar) pre-coated with Matrigel matrix (BD Biosciences). While NCTC1469 cells (2 × 10^5^ cells/mL) were plated bottom chambers and maintained in 500 μL DMEM medium supplemented with 10% FBS. After co-culture for 24 h or 48 h, the medium was discarded, pre-chilled PBS was washed twice, the non-migrating cells in the upper side were gently scraped off with a cotton swab, 500 μL of 4% paraformaldehyde was added to each small chamber. After 15 min, rinsed 3 times with pre-chilled PBS. Subsequently, immersed in 0.5% crystal violet staining solution and stained for 10-15 min. PBS washing was used to wash away the excess staining solution, and microscopic observation was carried out while keeping the membrane of the chambers slightly moist. 400 × objective microscope was used to examine and capture the cells, and three photographs of different areas were fixedly selected for each chamber, and the cells in each area were counted as the standard for data processing. We quantified the percentage of invading cells utilizing the ImageJ software. Each experiment was repeated in triplicate.

### Animal

#### Experimental models and drug administration

6–8 weeks old male BALB/c mice (n = 30) were purchased from Qinglongshan Animal Breeding Farm, Jiangning District, Nanjing City (license No: SCXK (Su) 2017 − 0001). All mice were fed in a specific-pathogen-free condition, same as "2.4" (Ethic Committee approval No. 201906A042). To evaluate the inhibitory efficacy of AC on liver metastasis in colon cancer in vivo, As in our previous studies, we performed the orthotopic transplantation colon cancer model to assess liver metastasis [24]. Briefly, CT26.WT cells (1 × 10^6^ cells/mL) were injected into the axilla (6 mice). These mice were sacrificed ten days after injection (the tumor size grew to about 1cm^3^), these tumors were removed and chopped into 1mm^3^ debris. Then, the left lower abdomen of the mice was incised and the tumor debris was attached to the cecum colonic area of the mice using Histoacryl adhesive. Finally, the abdominal cavity of the mice was sutured. The identical surgical procedure was followed for the Sham group (n = 6), except for tumor attachment.

Eighteen mice were arbitrarily allocated into three groups (n = 6): the experimental model group, the 5-Fu treatment group, and the AC treatment group (at the most effective dose of our previous studies). Each group was administered the corresponding dosage of drugs for 15 days. Whereas, physiological saline at the same dose was given to the sham and the model group. The 5-Fu group was administered via i.p. injection at 30 mg/kg/3d every other day. The AC was received intragastric administration at 6 g/kg/d every day. The body mass of each group of mice was recorded every three days.

#### Sample collection

On day fifteen, mice were anesthetized with 2% isoflurane one hour after the last dose, and whole blood was obtained from the orbital sinus. All serum samples were then collected at 3,000×*g* centrifugal speed for 10 min and stored at − 80 °C until later experiments. The number of metastatic liver tumor nodules was evaluated and counted during the abdominal anatomy conducted. The liver, tumor, spleen, and thymus were extracted, weighed, and recorded. Samples for histological examination were stored in 4% neutral paraformaldehyde, and those for tissue homogenization were stored in liquid nitrogen.

The tumor inhibition ratio (TIR), Metastasis rate (MR), and spleen or thymus index were calculated by the following formulas: Tumor Inhibition ratio (TIR) (%) = (1–mean tumor weight of the AC treated group/mean tumor weight of the model group) × 100%; Liver Metastasis ratio (MR) (%) = (average number of metastases in the AC treated group/average number of metastases in the model group) × 100%; Spleen or thymus index (mg·g^−1^) = (spleen weight or thymus weight/body weight) × 100%.

#### Histological analysis

Preparation of liver tissue samples for routine hematoxylin and eosin (H&E) to observe and evaluate the morphological features of liver metastasis from colon cancer cells. Mice were sacrificed with isoflurane, and the liver was surgically extracted. Subsequently, the whole liver was fixed 48 h with 10% formalin. Following dehydrated in alcohol and embedded with paraffin, and routine H&E staining for histopathology. Histological sections were photographed utilizing an IX51 microscope (Olympus Corporation, Japan). The pathological image was measured and analyzed with ImageJ software.

### ELISA assay

The expression levels of pro-inflammatory factors CXCL8 (MBE10287), IL1-β (MBE10289), TNF-α (MBE10037), IFN-γ (MBE10182), and IL-6 (MBE10288) of mice serum samples and the cell supernatants were determined with ELISA Quantitation kit (Jiancheng, Nanjing, China). Related ELISA steps are carried out based on the instructions provided by the manufacturer.

### qRT-PCR analysis

RNAiso plus kit (Vazyme, Nanjing, China) was utilized to extract total RNA from CT26.WT cells, tumors, and liver tissues of mice, respectively. 500 ng of the total RNA was synthesized into the first cDNA by employing a reverse transcription kit (PrimeScript™ RT). cDNA amplified was conducted with SYBR Green^®^ Premix Ex Taq™ II. PCR temperature cycling conditions with the following: 95 °C for 30 s, then 40 cycles of 95 °C for 10 s, 30 s at 60 °C, 95 °C for 15 s, and 60 s at 60 °C. The final extension: 95 °C for 15 s. The mRNA levels of CXCL8, CXCR2, CXCR1, PI3K, and AKT, and E-cadherin, N-cadherin, vimentin, snail in tissue samples and colon cancer cells were accessed. The expression level of GAPDH was programmed as the reference, and the data were used for calculation by the 2^−ΔΔCt^method. The primers are showed in Additional file 1: Table S3.

### Western blotting analysis

Cultured CT26.WT cells, tumors or liver tissues lysed in RIPA buffer with PMSF, proteinase inhibitors and phosphatase inhibitors (Sigma-Aldrich) to yield the homogenate. Following the total protein levels were quantified via the BCA protein assay kit (Thermo Fisher Scientific, USA). Equivalent amounts of 40 µg protein extracts were loaded and electrophoresed with 10% SDS-PAGE, and then transferred unto the polyvinylidene fluoride (PVDF) membranes. Next, after blocking with 3% BSA, the membranes were incubated overnight with the corresponding primary antibodies, subsequently washing and incubating with secondary antibodies (goat anti-rabbit). Finally, immunoreactivity was scanned with an enhanced chemiluminescence system. All experiments were conducted out in triplicate independently.

### Statistical analysis

All data were calculated utilizing t-test to calculate statistical differences between the two groups and one-way ANOVA for the more groups, using GraphPad Prism 8 software. *P* < 0.05 Differences was defined as significant, very/extremely significant when *P* < 0.01 and *P* < 0.001. Quantitative statistical was uniformly conducted as "mean ± se".

## Results

### Prediction and enrichment analysis results of potential therapeutic targets and pathways in LMCC

**Fig. 1:**
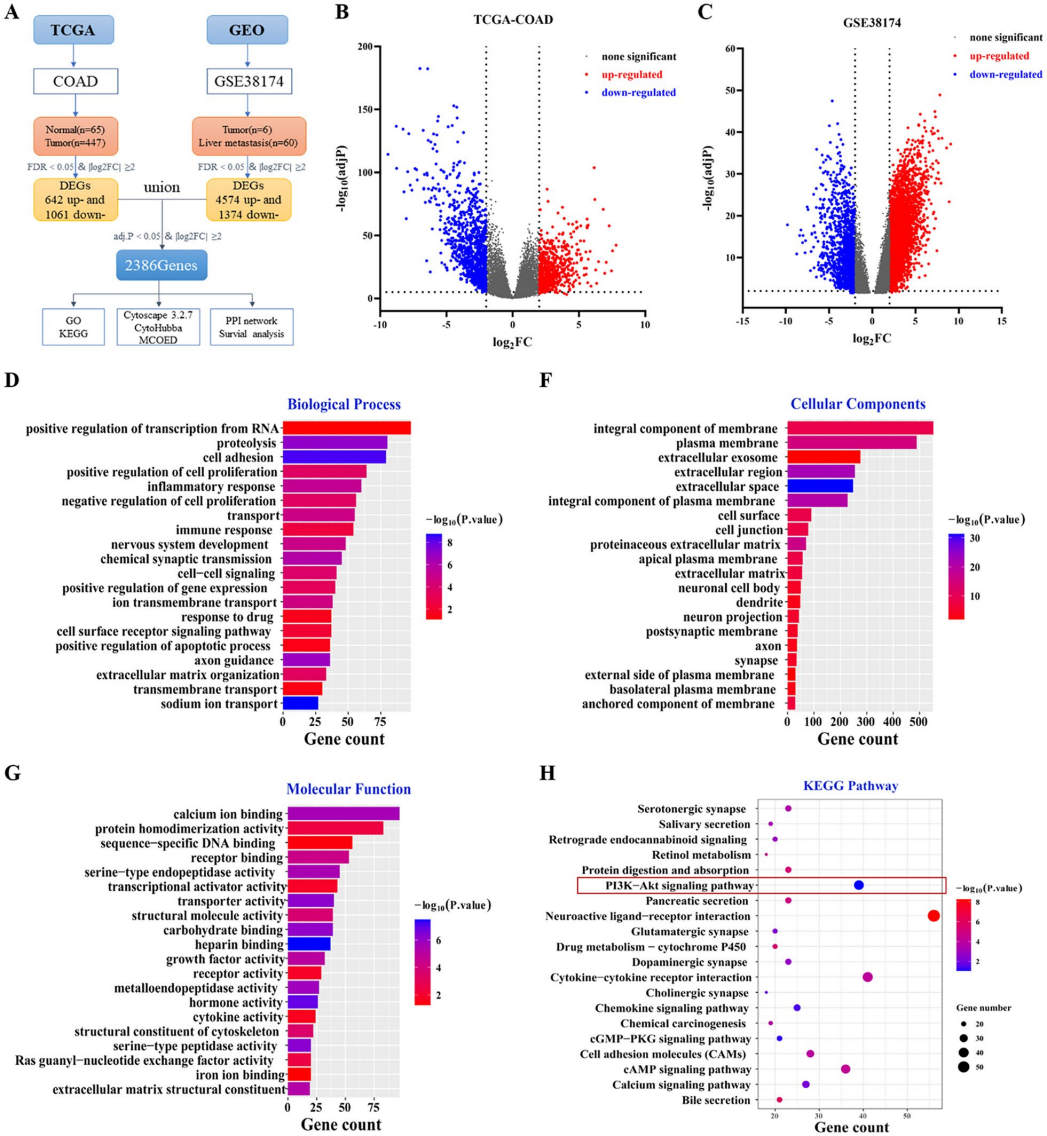
Prediction and enrichment analysis results of targets and pathways. **A** The flowchart of Bioinformatics analysis. **B** Volcano map of DEGs in COAD. **C** Volcano map of DEGs in GSE38174. **D**–**G** GO enrichment analysis of 2386DEGs. **H** KEGG pathway analysis of 2386DEGs

The flowchart of Bioinformatics analysis in this research as shown in Fig. 1A. The COAD (Colon adenocarcinoma) dataset downloaded from the TCGA database contained 447 colon cancer tissues and 65 normal tissues. 6 normal colon tissue samples and 60 liver metastases tissues samples from colon cancer were acquired in the GSE38174 datasets obtained from GEO database. We took the differentially expressed genes when |log_2_ FC|≥2 and *p* < 0.05 for statistics. At last, 2386DEGs (encompassing 1162 up-regulated genes and 1224 down-regulated genes) are tightly connected to LMCC obtained from “Limma” package of R (Fig. 1B–C). To further investigate the mechanism of LMCC, 2386DEGs were selected for GO enrichment analysis, which includes biological process, cell components, and molecular functions (Fig. 1D–G) and KEGG pathway analysis (Fig. 1H). GO annotation analysis revealed that the main biological processes in which DEGs participated were RNA transcriptional regulation, cell adhesion, cell proliferation, inflammatory response, immune response, cell-cell signaling, and cell surface receptor signaling. KEGG pathway analysis enriched 56 terms and the Neuroactive ligand-receptor interaction pathway, Cytokine-cytokine receptor interaction, PI3K/Akt signaling pathway, cAMP signaling pathway, Cell adhesion molecules (CAMs), Chemokine signaling pathway were remarkably enriched (*p* < 0.05). Previously, research found that immune-related neuroactive ligand-receptor interaction pathways can influence the interactions between microenvironmental cells and tumor cells [26]. Activation of inflammation-related cytokine-cytokine receptor interactions integrates with multiple signaling pathways to modulate cancer initiation and progression [27]. According to literature reports, the PI3K/Akt signaling pathway modulates critical normal cellular activities that are required for cancer and metastasis, including cell survival, proliferation, cell cycle regulation, angiogenesis, and metabolism [28, 29], which was quite similar to the biological processes of GO functional analysis. Aberrations within PI3K/Akt signaling pathway were frequent occurrences in solid tumors and metastasis, particularly in colon cancer [30]. These results suggested that the Neuroactive ligand-receptor interaction, Cytokine-cytokine receptor interaction and PI3K/Akt signaling pathway may be responsible for an important contribution in the mechanism of LMCC.

**Fig. 2:**
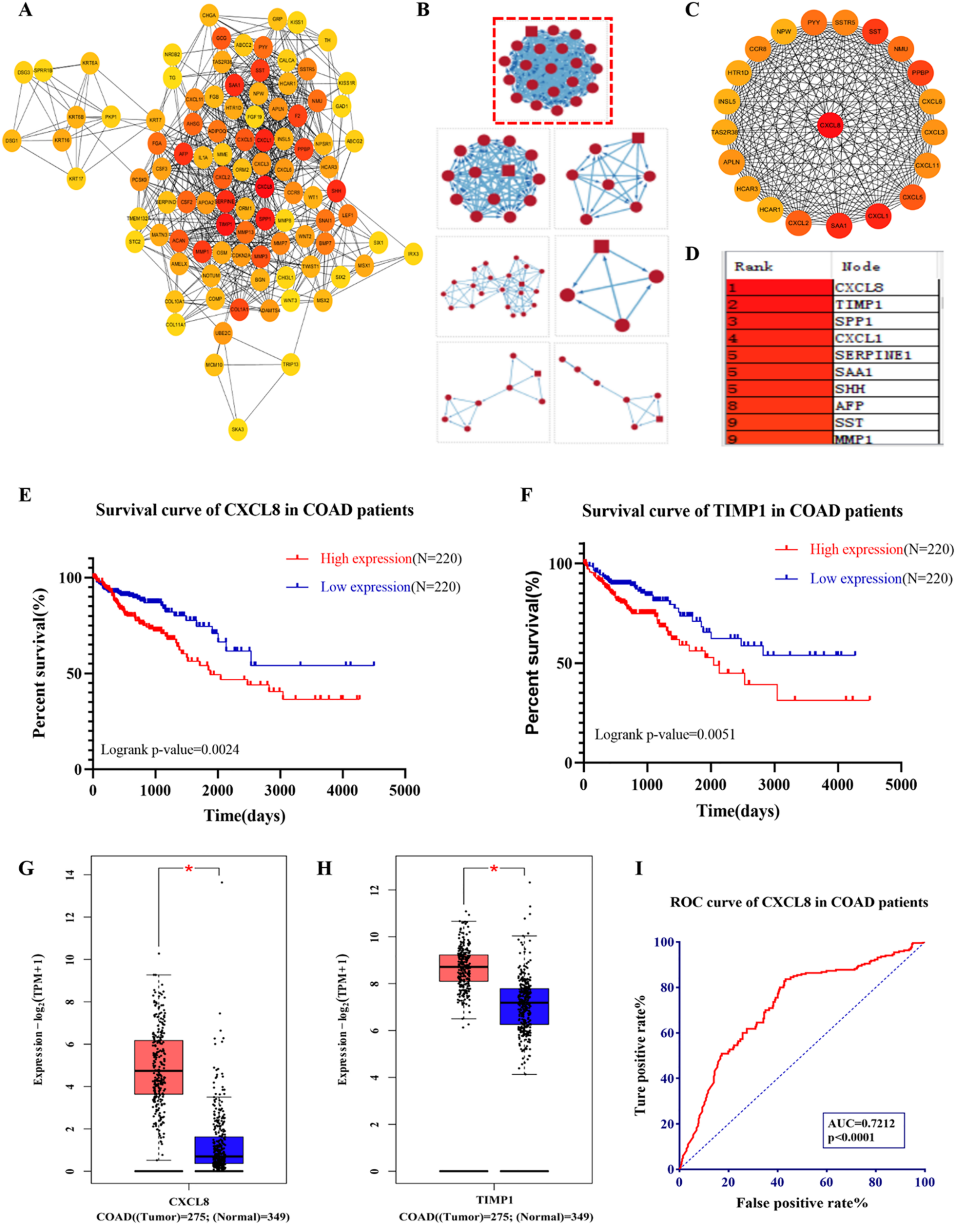
Construction and analysis of DEGs interaction network. **A** PPI network of TOP 100 significantly differentially expressed RNAs. **B**–**D** Hub modules and top genes. **E**–**F** KM survival analysis of hub genes expression in colon cancer patients. **G**–**H** The expression levels of CXCL8 and TIMP1 in COAD patients. **I** ROC curves of CXCL8 in COAD patients

To further identify the hub gene in differentially expressed genes, a PPI network of these significant DEGs was obtained from the STRING website and visualized using Cytoscape3.7.2 software, including 590 nodes and 2367 edges. Then, we used the CytoHubba plug-in to analyze the connectivity of each node in the PPI network based on degree value, the top 100 nodes in the network of connectivity are shown in Fig. 2A. Besides, the MCODE plug-in was applied to compute the modules in the network with particular functionality. Module 1, which received the highest score of 20.00, contains 21 nodes and 210 edges. The top ten hub genes are CXCL8, TIMP1, SPPI, CXCL1, SERPINE1, SAA1, SHH, AFP, SST, MMP1 (Fig. 2B–D). Moreover, KM survival analysis results showed that the hub genes with significant influence on overall survival in COAD patients were CXCL8 and TIMP1 (*p* < 0.01), and patients with low CXCL8 and TIMP1expression have better survival (Fig. 2E–F). Meanwhile, A box plot showed significantly increased expression of CXCL8 and TIMP1 in TCGA COAD samples (n = 275) as compared to the normal colon samples (match TCGA normal and GTEx data, n = 349) (*p* < 0.01) (Fig. 2G–H). In addition, the area under the curve (AUC) value for CXCL8 was 0.7212 (95% confidence interval: 0.6777 to 0.7647, *p* < 0.0001) (Fig. 2I), which means that CXCL8 could be regarded as a better prognostic biomarker for colon cancer patients. And it has been reported that CXCL8 and its receptors do play an important role in colorectal liver metastasis [31]. CXCL8 and its receptors contributed to the survival, proliferation, migration and invasion of circulating tumor cells, in addition to promoting colorectal cancer liver metastasis through the induction of EMT in cancer cells.

### AC serum inhibits the proliferation and metastasis ability of CT26.WT Cells

**Fig. 3:**
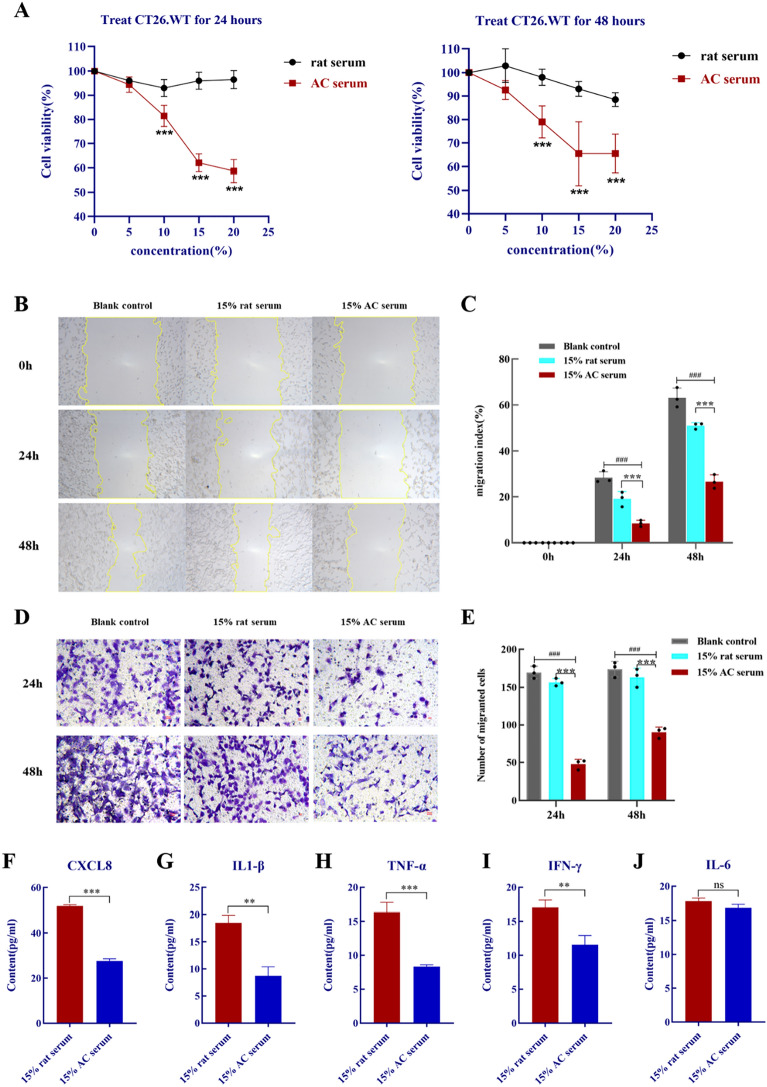
AC suppressed the growth, migration and invasion ability of CT26.WT cells. **A** In CCK8 experiments, AC inhibited CT26.WT cell proliferation in a concentration-dependent manner. **B**–**C** AC inhibited the mobility of CT26.WT cells in wound-healing assay. **D**–**E** AC inhibited invasion of CT26.WT cells. Scale bar: 20 μm. **F**–**J** The content of CXCL8, IL1-β, TNF-α, IFN-γ and IL-6 in the cell culture supernatant were detected by ELISA (n = 3). *p* < 0.01 (^**##**^) and *p* < 0.001(^**###**^), compared with the blank control group; *p* < 0.05 (*), *p* < 0.01 (**) and *p* < 0.001(***), compared with the rat serum group

Firstly, CCK8 assay results showed that AC serum decreased CT26.WT cells viability concentration-dependently compared with the rat serum group of equal concentration (*p* < 0.001), and the rat serum treated group were no significant influence on the cell viability compared to the blank control group (the administration of serum concentration regarded as 0) **(**Fig. 3A). Because of CCK8 assays presented that AC serum concentrations between 15 and 20% had a similar rate of suppression of CT26.WT cells, so we chose a 15% concentration of AC serum for the following experiments. In migration assay, the wound area in the AC treated group was greater than the AC-free group, but there was no significant difference between the blank control and rat serum group, suggested that AC treatment could independently and significantly inhibit the migration ability of CT26.WT cells (Fig. 3B–C). Similar effects were also discovered in transwell invasion assay that AC serum greatly decreased invasion ability of CT26.WT cells (Fig. 3D–E**)**. These results indicated that AC serum markedly inhibited the viability and possessed an anti-metastasis function on colon cancer cells.

According to the results of DEGs interaction network and survival analysis, CXCL8 may be critical in the development and prognosis of colon cancer. As a secreted-type cytokine, CXCL8 is a nearly undetectable molecule in the physiological environment, but it is activated rapidly by a variety of factors that contribute to tumorigenesis and inflammatory processes, including TNFα, IL-1β, IFN-γ, ROS, etc.[32, 33]. Therefore, we wondered whether AC regulates the expression of CXCL8 and related pro-inflammatory factors in cell supernatant. Results of ELISA detection shown in Fig. 3F–J, the levels of CXCL8 and pro-inflammatory factors TNFα, IL-1β, IFN-γ were significantly down-regulated after AC serum administration (*p* < 0.01). Noteworthy, we also detected IL6, which has a similar function to CXCL8, but the difference in expression after AC treatment was not significant (*p* > 0.05). Taken together, the AC serum effectively relieved the indicators of inflammation and down-regulating the expression of colon cancer biomarker CXCL8 as determined by bioinformatics analysis but has no impact on another pro-inflammatory chemokine IL6. The results were highly concordance with those obtained from bioinformatics analysis.

### AC suppresses the tumor growth of CC-orthotopic transplantation mice model

**Fig. 4:**
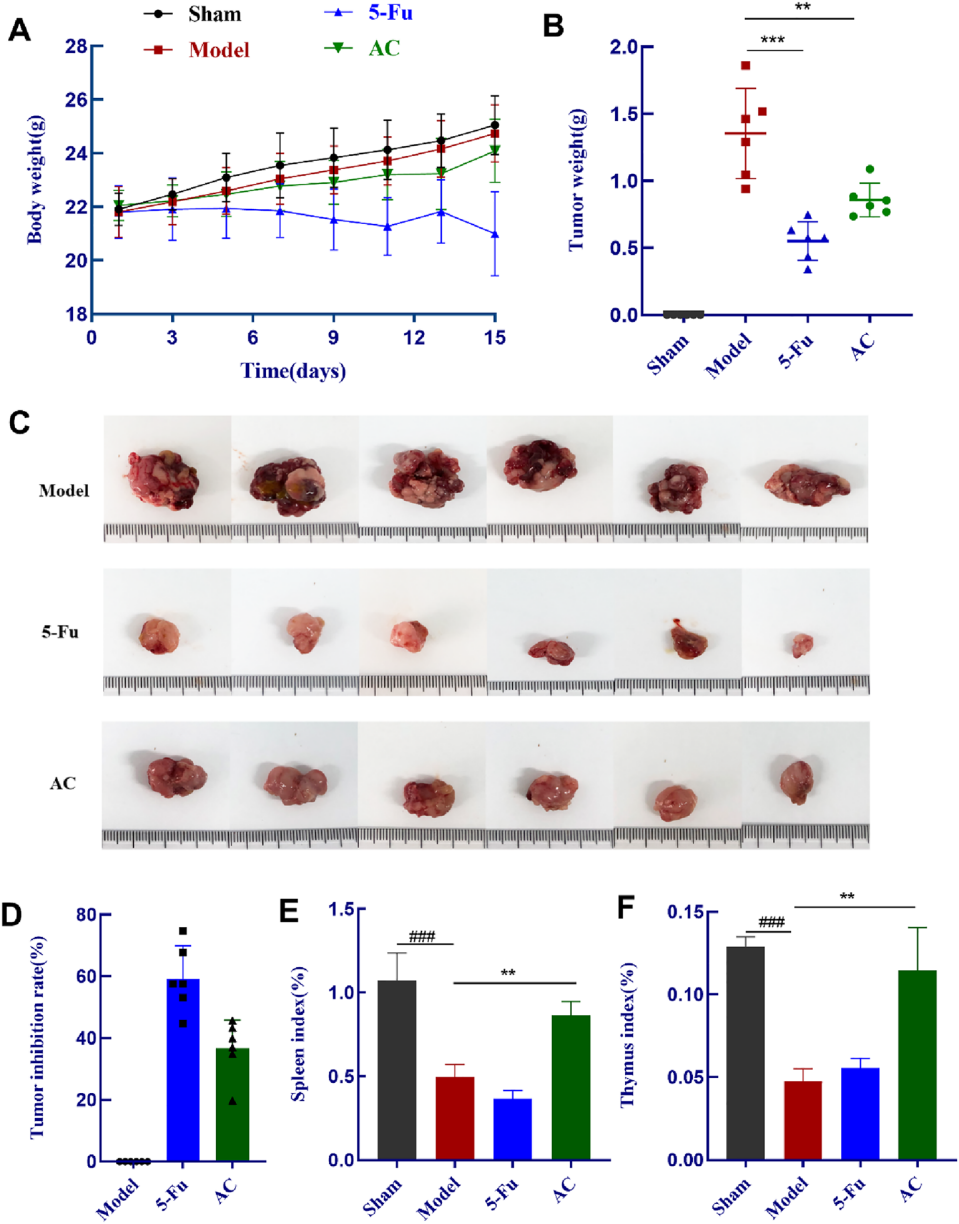
AC suppressed growth of orthotopic transplantation tumors (n = 6). **A** Body weight were recorded every three days during the treatment; **B** Tumor weight of mice in each group; **C** Images of solid tumors in each group; **D** Tumor inhibition rate; **E**–**F** Spleen/Thymus index of mice in each treated group. *p* < 0.01(^**##**^) and *p* < 0.001(^**###**^), compared with the sham group; *p* < 0.05(*), *p* < 0.01(**) and *p* < 0.001(***), compared with the model group

In this experiment, the colon cancer orthotopic transplantation model mice had a success rate of 100%. 5-Fluorouracil (5-Fu) is an essential chemotherapy drugs widely used to treat colon cancer patients in the standard first-line treatment for several decades and has been employed as a positive control drug in preclinical animal studies [34]. The abdomens of the model mice showed progressive swelling, accompanied by diarrhea, reduced activity, and decreased intake of water and food. The mice given AC had a higher quality of life than the model group, with shiny and smooth back hair, normal behavior, and increased water and food intake, while the 5-Fu treated group were in worse mental status and sluggish movements. The body weight change curves of mice under the treatment period are shown in Fig. 4A, and results revealed that AC has no discernible toxicity or adverse effects and significantly improved the behavioral state, hunger, and gastrointestinal reactivity of colon cancer model mice. Meanwhile, as compared with the model group, model mice of the 5-Fu and AC treated groups had considerably lower tumor weights (*p* < 0.01) (Fig. 4B–C). The 5-Fu group inhibited tumors at a rate of 59.22 ± 0.11%, and the AC group showed 36.63 ± 0.09% (Fig. 4D). Interestingly, mice in the AC treated groups had a stronger spleen and thymus index compared to the model group (*p* < 0.01) (Fig. 4E–F). These results demonstrated that AC could be an effective anticancer agent in the colon cancer orthotopic-transplanted model mice, and it could improve the spleen and thymus indices to recover the immune organ function to a certain extent.

### AC reduced liver metastasis in CC-orthotopic transplantation mice model

**Fig. 5:**
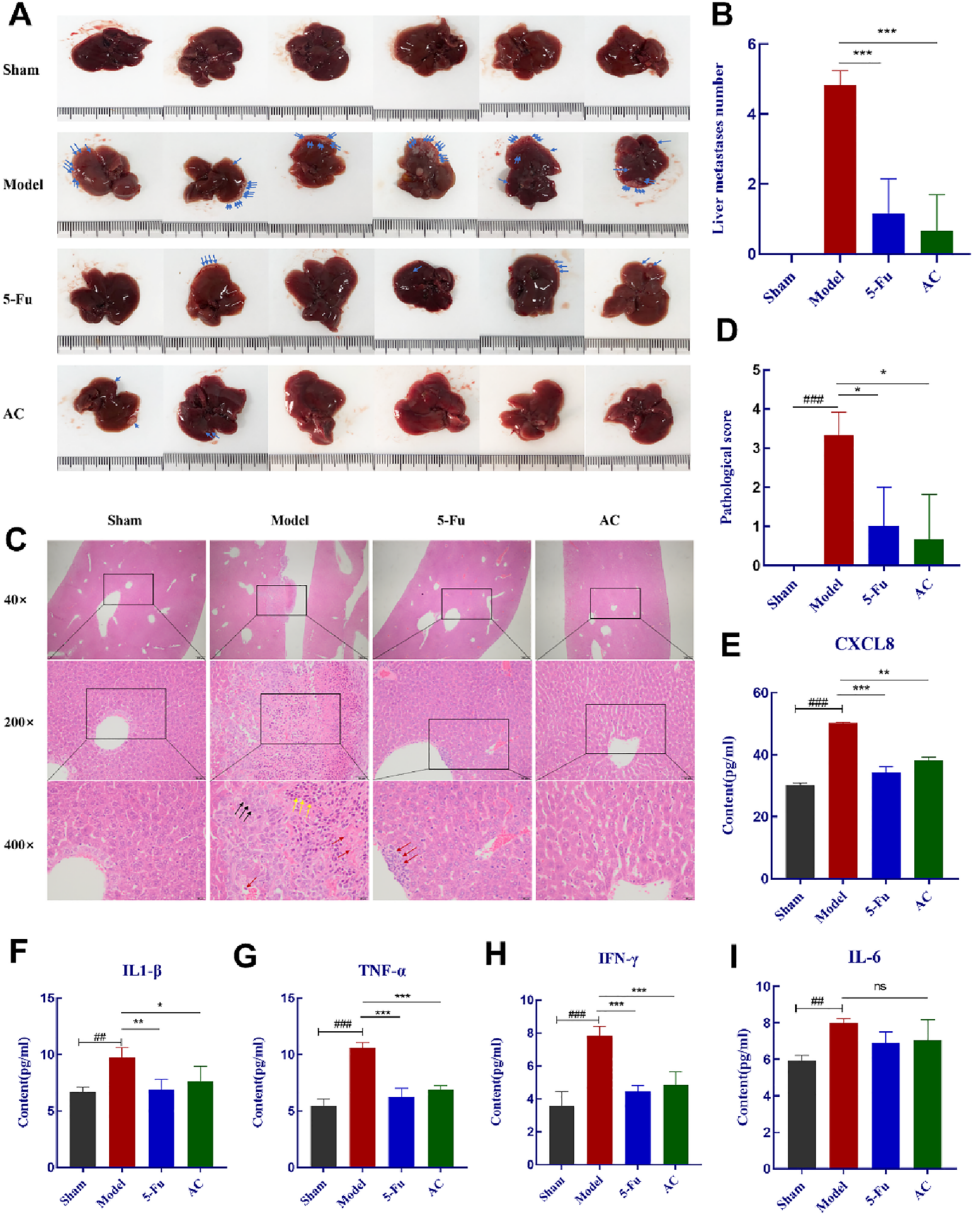
AC reduced liver metastasis in CC-orthotopic transplantation mice model (n = 6). **A** Images of liver metastasis foci in each group of model mice (blue arrows); **B** Number of hepatic metastasis of mice after drug administration; **C**–**D** Liver metastasis histopathological changes (**H**&**E** staining) and pathological score. tumor cell clusters (dark arrow), hepatocyte cord arrangement disorder (yellow arrow) and inflammatory cell infiltration (red arrow). **E**–**I** The content of CXCL8, IL1-β, TNF-α, IFN-γ and IL-6 in serum was detected with ELISA. *p* < 0.05(^**#**^), *p* < 0.01(^**##**^) and *p* < 0.001(^**###**^), compared with the sham group; *p* < 0.05(*), *p* < 0.01(**) and *p* < 0.001(***), compared with the model group

The abdominal autopsy revealed that metastatic foci with apparently single or numerous white nodules were observed in the liver of the model mice **(**blue arrows, Fig. 5A). While the number of metastases foci on the liver were significantly decreased in the 5-Fu or AC treated group (*p* < 0.01) (Fig. 5B). Following that, H&E staining was utilized to assess morphological alterations of liver tissue of colon cancer model mice in each group (Fig. 5C–D). Compared to the sham group, the model group presented distinct infiltration of tumor cells characterized by atypicality, different dimensions, dense arrangement and irregularity. Additionally, a large number of hepatocyte nuclei and cytoplasm are occupied by tumor cell clusters (dark arrow). Meanwhile, hepatocyte cord arrangement disorder (yellow arrow) and inflammatory cell infiltration (red arrow) also appeared. The histological alterations in liver tissue were improved after 5-Fu or AC treatments. Likewise, we detected the colon cancer prognostic biomarker CXCL8 and its associated pro-inflammatory cytokines (IL1-β, TNF-α, IFN-γ and IL-6) in the serum of mice in each group. The ELISA results were consistent with our in vitro cell experiments. After AC administration, the expression levels of inflammatory cytokines were significantly down-regulated, especially the levels of CXCL8, TNF-α and IFN-γ (*p* < 0.01) (Fig. 5E–I). These results exhibited that AC could attenuate the inflammation indicators and inhibit liver metastasis in CC-orthotopic transplantation mice model.

### AC regulated the CXCL8/PI3K/AKT pathway in Colon cancer cells and orthotopic transplantation mice model

Results from PPI network visualization analysis indicated that CXCL8 has the best centrality in the PPI network constructed by DEGs and highly associated with LMCC. The hub gene CXCL8 is mainly enriched in Cytokine-cytokine receptor interactions pathway. The cytokine-cytokine receptor interactions have a crucial role in cellular communication through their specific receptor interactions [35]. Among these, inflammatory cytokine CXCL8 and its receptor CXCR1/2 axis have a confirmed regulatory role in pro-inflammation, cancer cell multiplication, invasion and metastasis [36]. Additionally, PI3K is the main intracellular downstream signal of CXCL8, PI3K causes Akt to undergo phosphorylation, and activated Akt may have a critical regulatory action in the cell survival, proliferation, angiogenesis, and metastasis of tumor cell [37, 38]. In practice, several experimental evidences have documented that high expression of CXCL8 binds to receptors CXCR1 and CXCR2, mediates the transcriptional process of PI3K/Akt/mTOR signaling cascade, and promotes metastasis in multiple malignant tumors [39, 40], specifically in colon cancer [36, 41]. Therefore, in combination with the positive effects of AC in inhibiting liver metastasis of colon cancer, it could be assumed that whether AC suppressed colon cancer progression and liver metastasis by modulating the CXCL8/PI3K/AKT signaling pathway, and we then validated in vivo and in vitro experiments respectively.

**Fig. 6:**
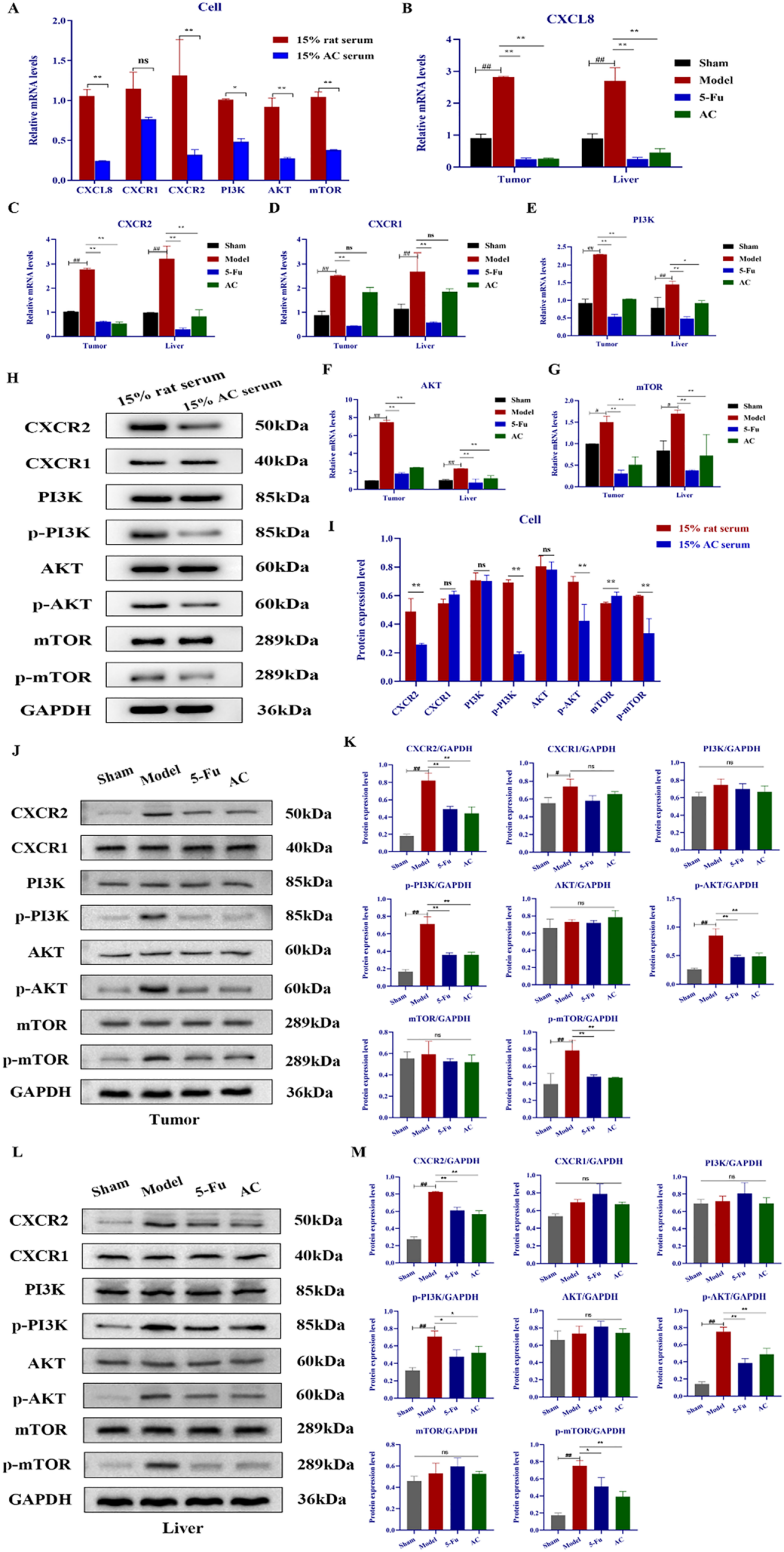
AC regulated the CXCL8/PI3K/AKT pathway in vivo and in vitro assays. **A**–**G** The influences of AC on the mRNA expression levels of CXCL8/PI3K/Akt pathway connected genes in colon cancer (n = 3). **H**–**M** The influences of AC on the protein expression of CXCL8/PI3K/AKT pathway associated proteins in colon cancer (n = 3) (**H**–**I**. CT26.WT cells; **J**–**K**. tumor tissues; **L**–**M**. liver tissues). *p* < 0.05 (^**#**^), *p* < 0.01(^**##**^), compared with the sham group; *p* < 0.05 (*), *p* < 0.01(**), compared with the model or rat serum group

Firstly, in vitro results illustrated that the mRNA levels of CXCL8, CXCR2, PI3K, AKT, and mTOR were significantly decreased in CT26.WT cells after 24 h of AC treatment (*p* < 0.01; Fig. 6A), but had no significant influence on the transcription levels of CXCR1. Then, we also detected the tumor tissue in situ and liver tissue of mice in each group. Consistently, results revealed that transcript levels of CXCL8, CXCR2 and PI3K, AKT, mTOR in vivo were substantially lowered in the 5-Fu and AC treated groups compared to the model group (*p* < 0.05; Fig. 6B–G). Noticeably, the significant down-regulation of the expression levels of CXCR2 proved that AC inhibited the binding of the chemokine CXCL8 to receptor CXCR2 but not CXCR1. In addition, western blotting analysis of chemokine receptor CXCR1 and CXCR2 protein expression showed similar trends to the corresponding mRNA levels both in vivo tissue and in vitro cellular specimens following AC or 5-Fu administration. The protein expression of CXCR2 and p-PI3K, p-AKT, and p-mTOR were decreased among the 5-Fu, AC water extract, and serum treated groups compared to the model or rat serum group (*p* < 0.05) (Fig. 6H–M). These results indicated that AC might be inhibiting colon cancer development and liver metastasis via inhibiting the CXCL8/CXCR2 axis and the PI3K/AKT/mTOR signaling pathway. Overall, the experimental results properly verify the above bioinformatics forecast.

### AC inhibited the EMT process of colon cancer

**Fig. 7:**
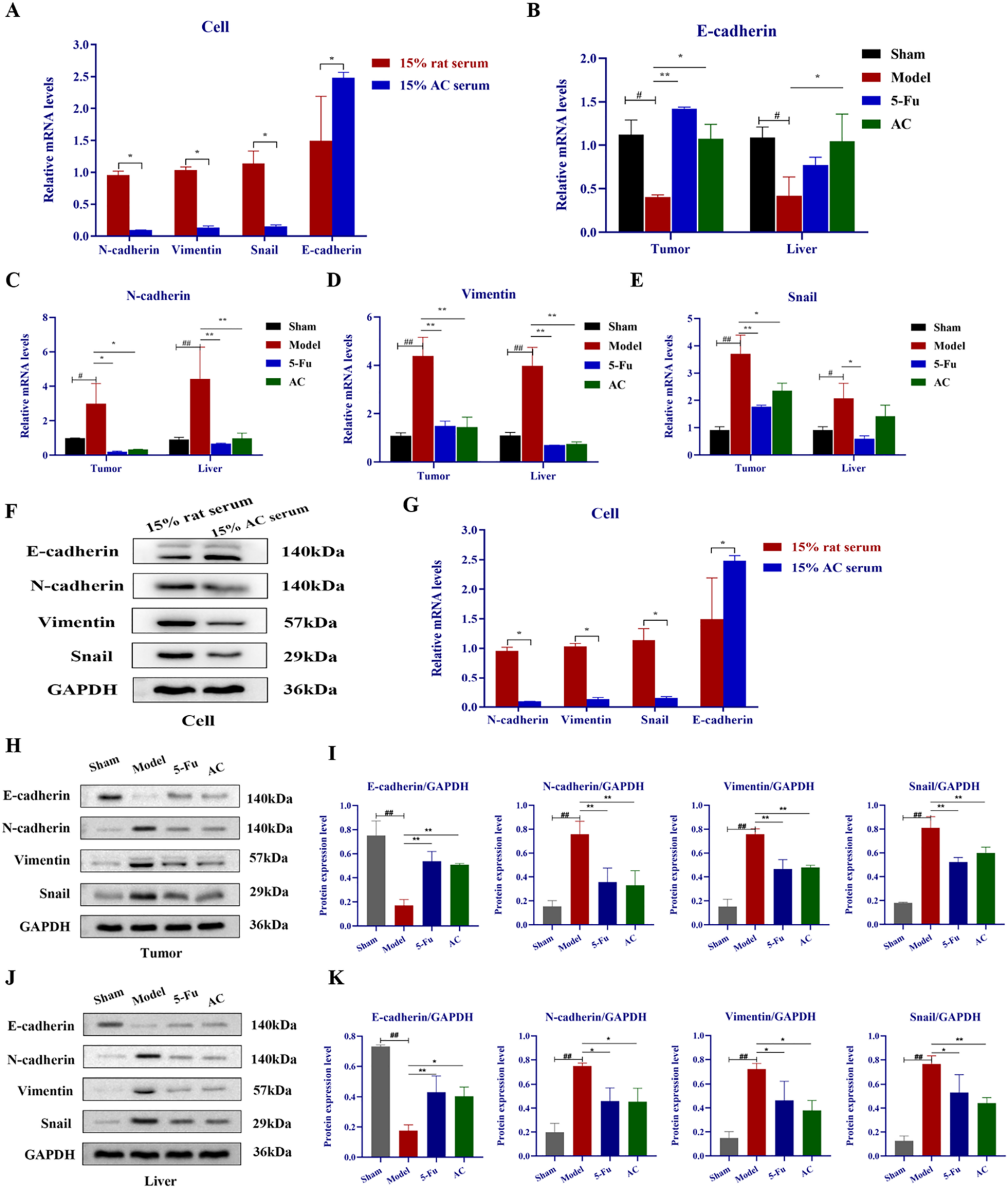
AC inhibited the EMT process in vivo and in vitro experiments. **A**–**E** The influences of AC on mRNA expression levels of EMT biomarkers in colon cancer by qRT-PCR (n = 3). **F**–**K** The influences of AC on the protein expression levels of EMT biomarkers in colon cancer detected by western blotting (n = 3) (**F**–**G**. CT26.WT cells; **H**–**I**. tumor tissues; **J**–**K**. liver tissues). *p* < 0.05 (^**#**^), *p* < 0.01(^**##**^), compared with the sham group; *p* < 0.05 (*), *p* < 0.01(**), compared with the model or rat serum group

In the light of the above functional GO analysis results, biological processes that DEGs participate in were mainly RNA transcription and cell adhesion, molecular functions tend to cytoskeleton composition, and cell area was primarily the cell membrane and surface, which correlate very well with the tumor metastasis related-EMT (Epithelial-mesenchymal transition) process. EMT is an essential dynamic biological process responsible for distant tumor metastasis, which occurs and enhances tumor cell migration and invasion when polarized tumor cells develop a mesenchymal phenotype [42, 43]. To examine the results of enrichment analysis, we attempted to illustrate the role of AC in EMT process by detecting the levels of typical EMT biomarkers (E-cadherin, N-cadherin, vimentin, snail). Likewise, qRT-PCR analysis revealed that the transcript levels of N-cadherin, vimentin and snail were significantly declined, while it evidently increased the E-cadherin levels after AC intervention in vivo and in vitro (*p* < 0.05) (Fig. 7A–E). Additionally, the protein levels of EMT markers were detected via western blotting, shown in Fig. 7F–K. The protein levels of N-cadherin, vimentin and snail were downregulated while the E-cadherin was upregulated significantly in AC treated group (*p* < 0.05). The experimentally verified results are in accordance with the predicted results. Taken together, our results suggested that AC might well be capable of suppressing tumor progression and liver metastasis in colon cancer via regulating the expression levels of EMT-related biomarkers.

## Discussion

Although several substantial breakthroughs in early diagnosis and therapy of colon cancer, the prognosis for patients remains poor [44]. Liver-tropic metastasis presents a daunting challenge to the treatment of colon cancer. Consequently, the underlying pathological mechanism of liver metastasis in colon cancer remains to be investigated [45]. Integrated Gene chips and bioinformatics analysis are emerging strategies for discovering differentially expressed genes and functional pathways that contribute to cancer pathogenesis [46, 47].

In the present study, we used TCGA COAD and GSE38174 datasets to screen the differentially expressed genes related to liver metastases of CC, and there were a total of 2386 DEGs identified. Subsequently, results of the combined KEGG and GO enrichment analysis, we discovered that the Cytokine-cytokine receptor interactions, PI3K/Akt signaling pathway and EMT process were closely connected to the mechanism of LMCC. In malignant colon tumors, the PI3K/Akt pathway is commonly exhibited as hyperactivated due to dysregulation of the DNA and transcription elements of the different signaling pathways [48, 49]. Due to this pathway is essential for cell survival, transcription, proliferation and growth processes, it is possible contributes to colon cancer increased aggressiveness and metastasis ability [50, 51]. EMT is another key process that drives colon cancer metastasis, which occurs during tumor growth and confers invasive and metastatic qualities on cancer cells [52, 53]. Interestingly, according to current study findings, the PI3K/Akt signaling pathway activation is a core regulatory mechanism controlling EMT in various malignancies. Aberrant hyper-activated PI3K/AKT pathway can inhibit the degradation of the downstream transcription factor snail and then attenuate the expression of E-cadherin, subsequently promoting the EMT and metastasis progression of tumor cells [54, 55]. Consequently, the results also confirm that our predictions are in accordance with those previously published in the literature.

Furthermore, we constructed a PPI network of DEGs to identify the hub gene. A core module was found to have the highest score using MCOED plug-in of Cytoscape. Then, we ranked genes on the basis of the degree of connectivity and screened out the hub gene CXCL8 in the module. Interestingly, compared to TCGA normal and GTEx data, CXCL8 was substantially expressed in colon cancer patients. According to the overall survival analysis results, while those CC patients with less CXCL8 expression have better overall survival. CXCL8, commonly known as IL-8, and its receptors CXCR1/2 are required for inflammatory cytokines activation and transportation, as well as tumor development and metastasis [32]. CXCR2 is the primary functional receptor binding to CXCL8 [56]. Previous research reports have shown that the combination of CXCL8 receptors CXCR1/2 promotes the progression of colon cancer and liver metastases [57, 58]. CXCL8 plays an essential regulatory role for cell motility, survival, angiogenesis, and proliferation in the tumor microenvironment and is also involved in regional anti-tumor inflammatory responses [59, 60]. In addition, CXCL8 has been shown to induce tumor related-EMT cascades through activation of the PI3K/AKT-ERK1/2 signaling pathway, which may also contribute to colon cancer cells resisting anoikis [61, 62]. Interestingly, PI3K has been demonstrated that perform as the primary intracellular signaling pathway downstream of CXCL8, promoting phosphorylation of its substrate, Akt, which is required for cell survival and migration [63]. In conclusion, the results of our bioinformatics analysis are in agreement with the literature studies. The CXCL8/CXCR1/2 chemokine axis and PI3K/AKT signaling pathway mediated EMT progress might well be a critical mechanism of colon cancer liver metastasis.

The therapeutic usage of chemoradiotherapy pharmaceutical therapies is severely limited due to the incidence of side effects and toxicities. As a result, alternative drugs that are very effective but have low adverse effects are required [64]. TCM has been reported to have suppressive effects majority of cancers and might even improve life quality and survival rates while exhibiting lower side effects than medications such as radiation and chemotherapy [65]. *Astragalus mongholicus* Bunge has been used in TCM for a long history in China. It was commonly used in combination with other herbs for the therapy of cancer patients [66, 67]. It is high in flavonoids, polysaccharides and saponins, which have anti-inflammatory, hypolipidemic, antioxidant, and immunostimulant efficacy against several types of malignancies [68, 69]. *Curcuma aromatica* Salisb. is another well-known TCM that is frequently combined with *Astragalus mongholicus* Bunge to treat various cancers. According to phytochemical studies, rhizomes of *Curcuma aromatica* Salisb. are abundant in curcuminoids, sesquiterpenes and monoterpenoids [70]. These chemical components have been proven to have a diverse range of biological functions, including anti-inflammatory, cytotoxic and antibacterial activity [71–73]. And our previous study demonstrated that the simultaneous use of *Astragalus mongholicus* Bunge and *Curcuma aromatica* Salisb. increased the fat-soluble ingredients content of *Curcuma aromatica* Salisb. in their combinatorial herbal formulations compared to single herbs, suggesting that there may be potential synergistic effects between the multiple bioactive constituents of AC [25]. Here, we proved that AC has favorable anti-tumor effectiveness against colon cancer at cellular or animal level. AC serum effectively decreased colon cancer cell growth, migration, and invasion ability. After AC intervention, the tumor weight and liver metastasis rate of CC-orthotopic model mice decreased significantly. At the same time point, AC could reduce the inflammation indicators in cell supernatant and mice serum, also up-regulated the spleen and thymus index in mice, suggesting that it has the potential to suppress tumor development and metastasis and recover the immune organ function.

We identified 13 main prototypical components in AC water extract and serum using UPLC-QQQ-MS/MS (Additional file 1: Table S2). The main components from *Astragalus mongholicus* Bunge are flavonoids, including (1) Calycosin-7-glucoside, (3) Ononin, (4) Calycosin, (5) Isomucronulatol 7-O-Glucoside, (6) Kaempferol, (7) Formononetin; and saponins components (8) Astragaloside IV, (9) Astragaloside I, (10) Astragaloside III. From *Curcuma aromatica* Salisb. are curcuminoids, such as (11) Curcumin, (12) Demethoxycurcumin, (13) Bisdemethoxycurcumin. Among them, Astragaloside IV, a quality control indicator for *Astragalus mongholicus* Bunge, inhibits the growth and metastasis of a variety of malignancies. Meanwhile, it controls EMT-related and autophagy-related pathways such as PI3K/AKT, Wnt/β-catenin and MAPK/ERK signaling pathways [74]. Calycosin inhibits proliferation, migration and invasion of breast cancer cells in a time and dose-dependent manner, and also suppresses the progression of EMT [75]. Ononin has an anti-inflammatory effect on lipopolysaccharide (LPS)-induced inflammation [76], and reported to inhibit proliferation of breast cancer cells via PI3K/AKT/mTOR signaling pathway [77]. Formononetin as anti-invasive agent for breast cancer, could inhibits migration and invasion of breast cancer cells by suppressing MMP-2 and MMP-9 through PI3K/AKT signaling pathways [78]. Curcumin is the most representative natural compound of *Curcuma aromatica* Salisb., especially in the treatment of a wide range of cancers [79]. As an adjuvant and complementary drug, Curcumin showed anticancer activity through inhibition of Wnt/β-catenin, Notch and PI3K/Akt/mTOR signaling pathways associated with colorectal cancer development [80]. Notably, the biological processes and signaling pathways involved in these compounds in the environment of cancer are in general accordance with the results of bioinformatic analysis in this work. It showed that the established UPLC-QQQ-MS/MS method was valid and reliable, and also provided a good basis for further research on the pharmacodynamic substance basis of AC against liver metastasis of colon cancer. In addition, 3 saponins components from *Astragalus mongholicus* Bunge were only found in the water extract, suggested that saponins components may be converted into other metabolic components after absorbed into the blood. It is also due to most Chinese herbal medicines must pass through the blood circulation before they can work [81], so we prepared AC-containing serum for in vitro cellular assays.

**Fig. 8:**
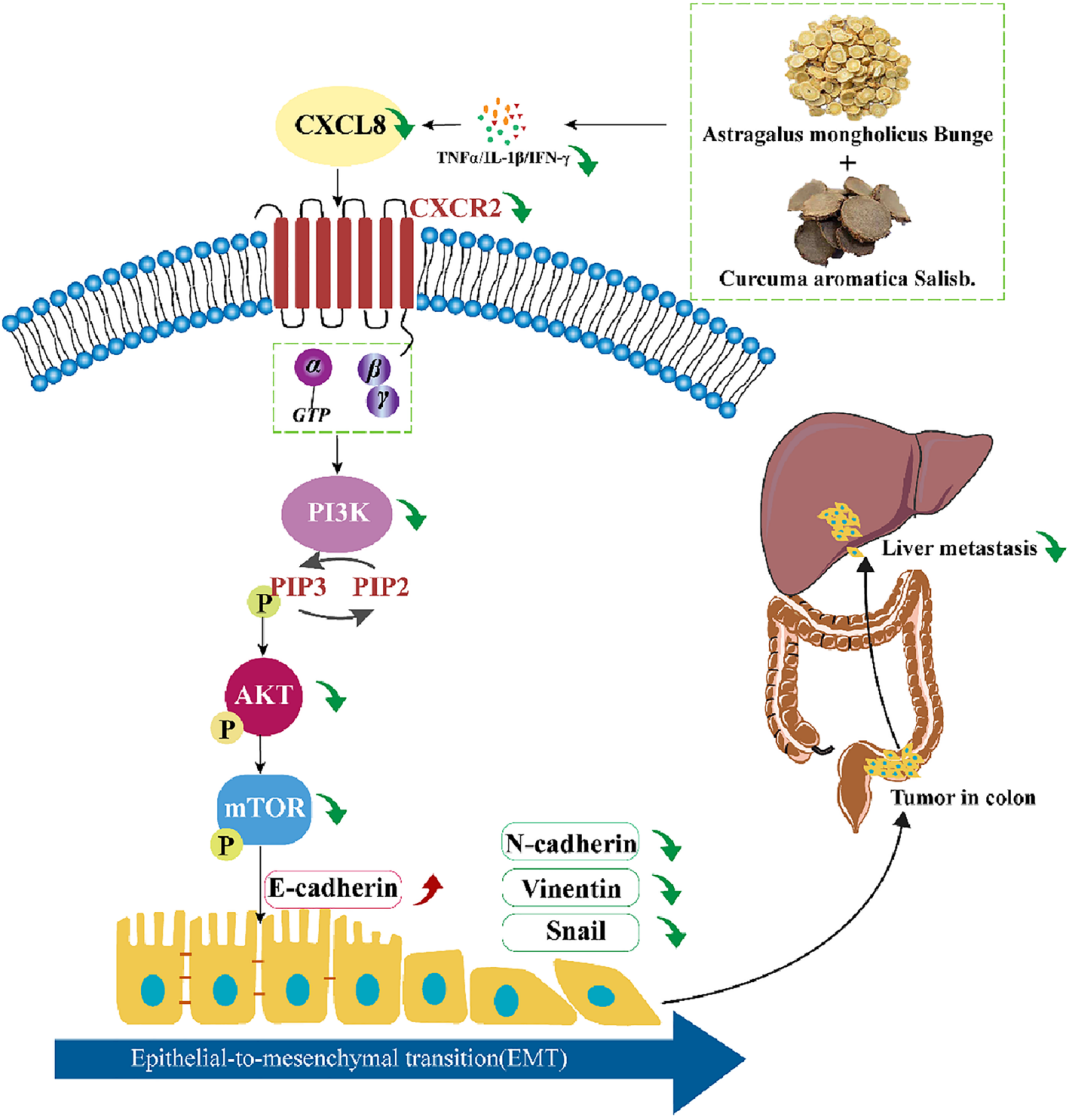
*Astragalus mongholicus* Bunge and *Curcuma aromatica* Salisb. inhibits liver metastasis of colon cancer by regulating EMT via the CXCL8/CXCR2 axis and PI3K/AKT/mTOR signaling pathway

Based on the results of bioinformatics analysis, we investigated whether AC could regulate the CXCL8/CXCR2 chemokine axis mediated PI3K/AKT and EMT signaling pathways. Firstly, in this research, ELISA assays cleared that CXCL8 was indeed highly expressed in cell supernatant and CC-orthotopic model mice serum. After AC treated, the expression levels of CXCL8 and its induced cytokines TNFα and IL-1β were significantly down-regulated. Then, western blotting and qRT-PCR results demonstrated that the proteins and mRNA levels of CXCR2 and p-PI3K, p-AKT, p-mTOR were significantly decreased in CT26.WT cells with AC treated for 24 h, the samples from in vivo assay also obtained similar results. Moreover, we proved that AC inhibited the binding of CXCL8 to receptor CXCR2 but not CXCR1. Finally, typical EMT markers N-cadherin, vimentin, and snail were significantly decreased, while the levels of E-cadherin were evidently increased after AC intervention in vivo and in vitro. Taken together, these results suggested that AC may down-regulate CXCL8/CXCR2 chemokine axis and suppressing the PI3K/Akt/mTOR pathway to inhibit the EMT process, thus acting on its anti-liver metastasis effect in colon cancer (Fig. 8).

However, there are also several unresolved issues in our study. In present study, we only identified the prototypical components of AC absorbed into the blood, further exploration of metabolic components and metabolic pathways of AC absorbed into the blood is needed, and we will continue use components or monomers for detailed studies. Furthermore, the exact mechanism of AC anti-metastatic activity against colon cancer liver metastasis remains inadequate, which warrants further investigation to confirm the mechanism using specific transgenic animals and mouse models of human-derived cells.

## Conclusion

In the present study, we screened the differentially expressed genes associated with liver metastasis in colon cancer and predicted the underlying signaling pathways of LMCC using bioinformatics methods. Then, the interaction network of DEGs was performed, and the hub gene was further clarified by extracting and analyzing the main regulatory network. Finally, in vitro and in vivo experiments, as well as a literature survey, were used to confirm the predicted results. In conclusion, our experimental evidence confirmed that AC may suppress the growth and liver metastasis in colon cancer by regulating EMT via the CXCL8/CXCR2 axis and PI3K/AKT/mTOR signaling pathway. These results showed that AC may be an effective formula against liver metastases of colon cancer from a potential pharmacological mechanistic perspective.

## Supplementary Information

**Additional file 1: Additional tables and figures. Table S1.** The MRM transitions and parameters of 13 compounds. **Table S2.** Regression equations and linear ranges of UPLC-QQQ-MS/MS. **Figure S1.** The total chromatograms of AC water extract and AC-containing serum with MRM mode. **Figure S2.** The chromatograms of AC water extract with the representative active ingredients. **Figure S3.** The chromatograms of AC-containing serum with the representative active ingredients. **Table S3**. Primer sequences involved in the experiment.

## Data Availability

The datasets used and/or analyzed during the current study are available from the corresponding author on reasonable request.

## Abbreviations

AC: *Astragalus mongholicus* Bunge—*Curcuma aromatica* Salisb
CRC: Colorectal cancer
CC: Colon cancer
RC: Rectal cancer
LMCC: Liver Metastasis of Colon cancer
TCGA: The Cancer Genome Atlas
GEO: Gene expression omnibus
COAD: Colon Adenocarcinoma
DEGs: Differentially expressed genes
GO: Gene ontology
KEGG: Kyoto Encyclopedia of Genes and Genomes
MCODE: Molecular Complex Detection
PPI: Protein–protein Interaction
GEPIA2: Gene Expression Profiling Interactive Analysis 2
GTEx: Genotype-Tissue Expression
EMT: Epithelial-mesenchymal transition
TCM: Traditional Chinese Medicine
ELISA: Enzyme-linked Immunosorbent Assay
qRT-PCR: Quantitative Real-Time PCR
PI3K: Phosphatidylinositol 3-kinase
AKT: Protein Kinase B
mTOR: The mammalian Target of Rapamycin

## References

[1] Sung H, Ferlay J, Siegel RL, Laversanne M, Soerjomataram I, Jemal A, Bray F (2021). Global Cancer Statistics 2020: GLOBOCAN estimates of incidence and mortality worldwide for 36 cancers in 185 countries. CA: A Cancer J Clin.

[2] Arnold M, Sierra MS, Laversanne M, Soerjomataram I, Jemal A, Bray F (2017). Global patterns and trends in colorectal cancer incidence and mortality. Gut.

[3] Siegel RL, Miller KD, Goding Sauer A, Fedewa SA, Butterly LF, Anderson JC, Cercek A, Smith RA, Jemal A (2020). Colorectal cancer statistics, 2020. CA: A Cancer J Clin.

[4] Zhu Y-J, Chen Y, Hu H-Y, Zhou Y-W, Zhu Y-T, Liu J-Y (2020). Predictive risk factors and online nomograms for synchronous colon cancer with liver metastasis. Front Oncol.

[5] Riihimäki M, Hemminki A, Sundquist J, Hemminki K (2016). Patterns of metastasis in colon and rectal cancer. Sci Rep.

[6] Liu J, Wang Y, Qiu Z, Lv G, Huang X, Lin H, Lin Z, Qu P (2021). Impact of TCM on tumor-infiltrating myeloid precursors in the tumor microenvironment. Front Cell Dev Biol.

[7] Wang K, Chen Q, Shao Y, Yin S, Liu C, Liu Y, Wang R, Wang T, Qiu Y, Yu H (2021). Anticancer activities of TCM and their active components against tumor metastasis. Biomed Pharmacother.

[8] Wang Y, Liu P, Fang Y, Tian J, Li S, Xu J, Zhao F, Yin X, Zhang Q, Li Y (2020). The effect of long-term traditional chinese medicine treatment on survival time of colorectal cancer based on propensity score matching: a retrospective cohort study. Evid Based Complement Alternat Med.

[9] Wang C-Y, Ding H-Z, Tang X, Li Z-G (2018). Comparative analysis of immune function, hemorheological alterations and prognosis in colorectal cancer patients with different traditional Chinese medicine syndromes. Cancer Biomark.

[10] Wu C-T, Tsai Y-T, Lai J-N (2017). Demographic and medication characteristics of traditional Chinese medicine users among colorectal cancer survivors: a nationwide database study in Taiwan. J Tradit Complement Med.

[11] Yin G, Tang D, Dai J, Liu MIN, Wu M, Sun YU, Yang Z, Hoffman RM, Li LIN, Zhang S, Guo X (2015). Combination efficacy of astragalus membranaceus and curcuma wenyujin at different stages of tumor progression in an imageable orthotopic nude mouse model of metastatic human ovarian cancer expressing red fluorescent protein. Anticancer Res.

[12] Tang D, Zhang S, Shi X, Wu J, Yin G, Tan X, Liu F, Wu X, Du X (2019). Combination of astragali polysaccharide and curcumin improves the morphological structure of tumor vessels and induces tumor vascular normalization to inhibit the growth of hepatocellular carcinoma. Integr Cancer Ther.

[13] Gu J, Sun R, Wang Q, Liu F, Tang D, Chang X (2021). Standardized Astragalus mongholicus Bunge-Curcuma Aromatica Salisb. extract efficiently suppresses colon cancer progression through gut microbiota modification in CT26-bearing mice. Front Pharmacol.

[14] Broderick P, Dobbins SE, Chubb D, Kinnersley B, Dunlop MG, Tomlinson I, Houlston RS (2017). Validation of recently proposed colorectal cancer susceptibility gene variants in an analysis of families and patients-a systematic review. Gastroenterology.

[15] Gao M, Zhong A, Patel N, Alur C, Vyas D (2017). High throughput RNA sequencing utility for diagnosis and prognosis in colon diseases. World J Gastroenterol.

[16] Zhao B, Baloch Z, Ma Y, Wan Z, Huo Y, Li F, Zhao Y (2019). Identification of potential key genes and pathways in early-onset colorectal cancer through bioinformatics analysis. Cancer Control.

[17] Guo Y, Bao Y, Ma M, Yang W (2017). Identification of key candidate genes and pathways in colorectal cancer by integrated bioinformatical analysis. Int J Mol Sci.

[18] Da Z, Gao L, Su G, Yao J, Fu W, Zhang J, Zhang X, Pei Z, Yue P, Bai B, Lin Y, Meng W, Li X (2020). Bioinformatics combined with quantitative proteomics analyses and identification of potential biomarkers in cholangiocarcinoma. Cancer Cell Int.

[19] Zhang C, Zhang G, Sun N, Zhang Z, Zhang Z, Luo Y, Che Y, Xue Q, He J (2020). Comprehensive molecular analyses of a TNF family-based signature with regard to prognosis, immune features, and biomarkers for immunotherapy in lung adenocarcinoma. EBioMedicine.

[20] Zhang L, Jiang X, Li Y, Fan Q, Li H, Jin L, Li L, Jin Y, Zhang T, Mao Y, Hua D (2020). Clinical correlation of Wnt2 and COL8A1 with colon adenocarcinoma prognosis. Front Oncol.

[21] Zhang R, Ye J, Huang H, Du X (2019). Mining featured biomarkers associated with vascular invasion in HCC by bioinformatics analysis with TCGA RNA sequencing data. Biomed Pharmacother.

[22] Nguyen D-D, Lee DG, Kim S, Kang K, Rhee J-K, Chang S (2018). Integrative bioinformatics and functional analyses of GEO, ENCODE, and TCGA reveal FADD as a direct target of the tumor suppressor BRCA1. Int J Mol Sci.

[23] Tang Z, Kang B, Li C, Chen T, Zhang Z (2019). GEPIA2: an enhanced web server for large-scale expression profiling and interactive analysis. Nucleic Acids Res.

[24] Sun R, Gu J, Chang X, Liu F, Liang Y, Yang X, Liang L, Tang D (2021). Metabonomics study on orthotopic transplantion mice model of colon cancer treated with Astragalus membranaceus-Curcuma wenyujin in different proportions via UPLC-Q-TOF/MS. J Pharm Biomed Anal.

[25] Yin G, Cheng X, Tao W, Dong Y, Bian Y, Zang W, Tang D (2018). Comparative analysis of multiple representative components in the herb pair Astragali Radix-Curcumae Rhizoma and its single herbs by UPLC-QQQ-MS. J Pharm Biomed Anal.

[26] Lvu W, Fei X, Chen C, Zhang B (2020). In silico identification of the prognostic biomarkers and therapeutic targets associated with cancer stem cell characteristics of glioma. Biosci Rep.

[27] Chen P-H, Yao H, Huang LJ-S (2017). Cytokine receptor endocytosis: new kinase activity-dependent and -independent roles of PI3K. Front Endocrinol.

[28] Laplante M, Sabatini DM (2012). mTOR signaling in growth control and disease. Cell.

[29] Vara JÁF, Casado E, De Castro J, Cejas P, Belda-Iniesta C, González-Barón M (2004). PI3K/Akt signalling pathway and cancer. Cancer Treat Rev.

[30] Zhu L, Derijard B, Chakrabandhu K, Wang B-S, Chen H-Z, Hueber A-O (2014). Synergism of PI3K/Akt inhibition and Fas activation on colon cancer cell death. Cancer Lett.

[31] Bie Y, Ge W, Yang Z, Cheng X, Zhao Z, Li S, Wang W, Wang Y, Zhao X, Yin Z, Li Y (2019). The crucial role of CXCL8 and its receptors in colorectal liver metastasis. Dis Markers.

[32] Ha H, Debnath B, Neamati N (2017). Role of the CXCL8-CXCR1/2 axis in cancer and inflammatory diseases. Theranostics.

[33] Cotton JA, Platnich JM, Muruve DA, Jijon HB, Buret AG, Beck PL (2016). Interleukin-8 in gastrointestinal inflammation and malignancy: induction and clinical consequences. Int J Interferon Cytokine Mediat Res.

[34] Van Cutsem E, Cervantes A, Adam R, Sobrero A, Van Krieken JH, Aderka D, Aranda Aguilar E, Bardelli A, Benson A, Bodoky G, Ciardiello F, Diaz-Rubio E, Douillard JY, Ducreux M, Falcone A, Grothey A, Gruenberger T, Haustermans K, Heinemann V, Hoff P, Köhne CH, Labianca R, Laurent-Puig P, Ma B, Maughan T, Muro K, Normanno N, Österlund P, Oyen WJG, Papamichael D, Pentheroudakis G, Pfeiffer P, Price TJ, Punt C, Ricke J, Roth A, Salazar R, Scheithauer W, Schmoll HJ, Tabernero J, Taïeb J, Tejpar S, Wasan H, Yoshino T, Zaanan A, Arnold D (2016). ESMO consensus guidelines for the management of patients with metastatic colorectal cancer. Annals Oncol.

[35] Han Z-J, Li Y-B, Yang L-X, Cheng H-J, Liu X, Chen H (2022). Roles of the CXCL8-CXCR1/2 axis in the tumor microenvironment and immunotherapy. Molecules.

[36] Lee YS, Choi I, Ning Y, Kim NY, Khatchadourian V, Yang D, Chung HK, Choi D, Labonte MJ, Ladner RD, Nagulapalli Venkata KC, Rosenberg DO, Petasis NA, Lenz HJ, Hong YK (2012). Interleukin-8 and its receptor CXCR2 in the tumour microenvironment promote colon cancer growth, progression and metastasis. Br J Cancer.

[37] Knall C, Worthen GS, Johnson GL (1997). Interleukin 8-stimulated phosphatidylinositol-3-kinase activity regulates the migration of human neutrophils independent of extracellular signal-regulated kinase and p38 mitogen-activated protein kinases. Proc Natl Acad Sci.

[38] Cheng GZ, Park S, Shu S, He L, Kong W, Zhang W, Yuan Z, Wang LH, Cheng JQ (2008). Advances of AKT pathway in human oncogenesis and as a target for anti-cancer drug discovery. Curr Cancer Drug Targets.

[39] Sun F, Wang J, Sun Q, Li F, Gao H, Xu L, Zhang J, Sun X, Tian Y, Zhao Q, Shen H, Zhang K, Liu J (2019). Interleukin-8 promotes integrin β3 upregulation and cell invasion through PI3K/Akt pathway in hepatocellular carcinoma. J Exp Clin Cancer Res.

[40] Zhai J, Shen J, Xie G, Wu J, He M, Gao L, Zhang Y, Yao X, Shen L (2019). Cancer-associated fibroblasts-derived IL-8 mediates resistance to cisplatin in human gastric cancer. Cancer Lett.

[41] Shuai F, Wang B, Dong S (2018). MicroRNA-204 inhibits the growth and motility of colorectal cancer cells by downregulation of CXCL8. Oncol Res.

[42] Wei C, Yang C, Wang S, Shi D, Zhang C, Lin X, Liu Q, Dou R, Xiong B (2019). Crosstalk between cancer cells and tumor associated macrophages is required for mesenchymal circulating tumor cell-mediated colorectal cancer metastasis. Mol Cancer.

[43] Ning X, Wang C, Zhang M, Wang K (2019). Ectopic expression of miR-147 inhibits stem cell marker and Epithelial-Mesenchymal Transition (EMT)-related protein expression in colon cancer cells. Oncol Res.

[44] Xie Y-H, Chen Y-X, Fang J-Y (2020). Comprehensive review of targeted therapy for colorectal cancer. Signal Transduct Target Ther.

[45] Sun H, Meng Q, Shi C, Yang H, Li X, Wu S, Familiari G, Relucenti M, Aschner M, Wang X, Chen R (2021). Hypoxia-inducible exosomes facilitate liver-tropic premetastatic niche in colorectal cancer. Hepatology.

[46] Alvarado G, Holland SR, Deperez-Rasmussen J, Jarvis BA, Telander T, Wagner N, Waring AL, Anast A, Davis B, Frank A, Genenbacher K, Larson J, Mathis C, Oates AE, Rhoades NA, Scott L, Young J, Mortimer NT (2020). Bioinformatic analysis suggests potential mechanisms underlying parasitoid venom evolution and function. Genomics.

[47] Zhou R, Wu K, Su M, Li R (2019). Bioinformatic and experimental data decipher the pharmacological targets and mechanisms of plumbagin against hepatocellular carcinoma. Environ Toxicol Pharmacol.

[48] Yu L, Wei J, Liu P (2021). Attacking the PI3K/Akt/mTOR signaling pathway for targeted therapeutic treatment in human cancer. Semin Cancer Biol.

[49] Tewari D, Patni P, Bishayee A, Sah AN, Bishayee A (2019). Natural products targeting the PI3K-Akt-mTOR signaling pathway in cancer: a novel therapeutic strategy. Semin Cancer Biol.

[50] Bishnupuri KS, Alvarado DM, Khouri AN, Shabsovich M, Chen B, Dieckgraefe BK, Ciorba MA (2019). IDO1 and kynurenine pathway metabolites activate PI3K-Akt signaling in the neoplastic colon epithelium to promote cancer cell proliferation and inhibit apoptosis. Cancer Res.

[51] Malinowsky K, Nitsche U, Janssen KP, Bader FG, Späth C, Drecoll E, Keller G, Höfler H, Slotta-Huspenina J, Becker KF (2014). Activation of the PI3K/AKT pathway correlates with prognosis in stage II colon cancer. Br J Cancer.

[52] Mizukoshi K, Okazawa Y, Haeno H, Koyama Y, Sulidan K, Komiyama H, Saeki H, Ohtsuji N, Ito Y, Kojima Y, Goto M, Habu S, Hino O, Sakamoto K, Orimo A (2020). Metastatic seeding of human colon cancer cell clusters expressing the hybrid epithelial/mesenchymal state. Int J Cancer.

[53] Rezaei R, Baghaei K, Amani D, Piccin A, Hashemi SM, Asadzadeh Aghdaei H, Zali MR (2021). Exosome-mediated delivery of functionally active miRNA-375-3p mimic regulate epithelial mesenchymal transition (EMT) of colon cancer cells. Life Sci.

[54] Wu X, Cai J, Zuo Z, Li J (2019). Collagen facilitates the colorectal cancer stemness and metastasis through an integrin/PI3K/AKT/Snail signaling pathway. Biomed Pharmacother.

[55] Wang S, Yan Y, Cheng Z, Hu Y, Liu T (2018). Sotetsuflavone suppresses invasion and metastasis in non-small-cell lung cancer A549 cells by reversing EMT via the TNF-α/NF-κB and PI3K/AKT signaling pathway. Cell Death Discov.

[56] Liu Q, Li A, Tian Y, Wu JD, Liu Y, Li T, Chen Y, Han X, Wu K (2016). The CXCL8-CXCR1/2 pathways in cancer. Cytokine Growth Factor Rev.

[57] Li J, Liu Q, Huang X, Cai Y, Song L, Xie Q, Liu F, Chen X, Xu P, Zeng F, Chu Y, Zeng F (2020). Transcriptional profiling reveals the regulatory role of CXCL8 in promoting colorectal cancer. Front Genet.

[58] Ogawa R, Yamamoto T, Hirai H, Hanada K, Kiyasu Y, Nishikawa G, Mizuno R, Inamoto S, Itatani Y, Sakai Y, Kawada K (2019). Loss of SMAD4 promotes colorectal cancer progression by recruiting tumor-associated neutrophils via the CXCL1/8–CXCR2 axis. Clin Cancer Res.

[59] Alfaro C, Sanmamed MF, Rodríguez-Ruiz ME, Teijeira Á, Oñate C, González Á, Ponz M, Schalper KA, Pérez-Gracia JL, Melero I (2017). Interleukin-8 in cancer pathogenesis, treatment and follow-up. Cancer Treat Rev.

[60] Waugh DJJ, Wilson C (2008). The interleukin-8 pathway in cancer. Clin Cancer Res.

[61] Shen T, Cheng X, Liu X, Xia C, Zhang H, Pan D, Zhang X, Li Y (2019). Circ_0026344 restrains metastasis of human colorectal cancer cells via miR-183. Artif Cells Nanomed Biotechnol.

[62] Cheng X-S, Li Y-F, Tan J, Sun B, Xiao Y-C, Fang X-B, Zhang X-F, Li Q, Dong J-H, Li M, Qian H-H, Yin Z-F, Yang Z-B (2014). CCL20 and CXCL8 synergize to promote progression and poor survival outcome in patients with colorectal cancer by collaborative induction of the epithelial–mesenchymal transition. Cancer Lett.

[63] Cheng J, Li Y, Liu S, Jiang Y, Ma J, Wan L, Li Q, Pang T (2019). CXCL8 derived from mesenchymal stromal cells supports survival and proliferation of acute myeloid leukemia cells through the PI3K/AKT pathway. FASEB J.

[64] Chen D, Zhao J, Cong W (2018). Chinese herbal medicines facilitate the control of chemotherapy-induced side effects in colorectal cancer: progress and perspective. Front Pharmacol.

[65] Xiang Y, Guo Z, Zhu P, Chen J, Huang Y (2019). Traditional Chinese medicine as a cancer treatment: modern perspectives of ancient but advanced science. Cancer Med.

[66] Shen L, Gwak SR, Cui ZY, Joo JC, Park SJ (2021). Astragalus-Containing Chinese herbal medicine combined with chemotherapy for cervical cancer: a systematic review and meta-analysis. Front Pharmacol.

[67] Lin S, An X, Guo Y, Gu J, Xie T, Wu Q, Sui X (2019). Meta-analysis of astragalus-containing traditional chinese medicine combined with chemotherapy for colorectal cancer: efficacy and safety to tumor response. Front Oncol.

[68] Liu C, Wang K, Zhuang J, Gao C, Li H, Liu L, Feng F, Zhou C, Yao K, Deng L, Wang L, Li J, Sun C (2019). The modulatory properties of Astragalus membranaceus treatment on triple-negative breast cancer: an integrated pharmacological method. Front Pharmacol.

[69] Bamodu OA, Kuo K-T, Wang C-H, Huang W-C, Wu ATH, Tsai J-T, Lee K-Y, Yeh C-T, Wang L-S (2019). Astragalus polysaccharides (PG2) enhances the M1 polarization of macrophages, functional maturation of dendritic cells, and T Cell-mediated anticancer immune responses in patients with lung cancer. Nutrients.

[70] Dosoky NS, Setzer WN (2018). Chemical composition and biological activities of essential oils of Curcuma species. Nutrients.

[71] Sultana S, Munir N, Mahmood Z, Riaz M, Akram M, Rebezov M, Kuderinova N, Moldabayeva Z, Shariati MA, Rauf A, Rengasamy KRR (2021). Molecular targets for the management of cancer using Curcuma longa Linn. phytoconstituents: a review. Biomed Pharmacother.

[72] Lee TK, Lee D, Lee SR, Ko Y-J, Sung Kang K, Chung SJ, Kim KH (2019). Sesquiterpenes from Curcuma zedoaria rhizomes and their cytotoxicity against human gastric cancer AGS cells. Bioorg Chem.

[73] Mishra S, Verma SS, Rai V, Awasthee N, Arya JS, Maiti KK, Gupta SC (2019). Curcuma raktakanda Induces apoptosis and suppresses migration in cancer cells: role of reactive oxygen species. Biomolecules.

[74] Chen T, Yang P, Jia Y (2021). Molecular mechanisms of astragaloside-IV in cancer therapy (Review). Int J Mol Med.

[75] Zhang Z, Lin M, Wang J, Yang F, Yang P, Liu Y, Chen Z, Zheng Y (2021). Calycosin inhibits breast cancer cell migration and invasion by suppressing EMT via BATF/TGF-β1. Aging (Albany NY).

[76] Dong L, Yin L, Zhang Y, Fu X, Lu J (2017). Anti-inflammatory effects of ononin on lipopolysaccharide-stimulated RAW 264.7 cells. Mol Immunol.

[77] Zhou R, Chen H, Chen J, Chen X, Wen Y, Xu L (2018). Extract from Astragalus membranaceus inhibit breast cancer cells proliferation via PI3K/AKT/mTOR signaling pathway. BMC Complement Altern Med.

[78] Zhou R, Xu L, Ye M, Liao M, Du H, Chen H (2014). Formononetin Inhibits migration and invasion of MDA-MB-231 and 4T1 breast cancer cells by suppressing MMP-2 and MMP-9 through PI3K/AKT signaling pathways. Horm Metab Res.

[79] Giordano A, Tommonaro G (2019). Curcumin and cancer. Nutrients.

[80] Villegas C, Perez R, Sterner O, González-Chavarría I, Paz C (2021). Curcuma as an adjuvant in colorectal cancer treatment. Life Sci.

[81] Bochu W, Liancai Z, Qi C (2005). Primary study on the application of Serum Pharmacology in Chinese traditional medicine. Colloids Surf B.

