# Supplementary material for: Astragalus mongholicus Bunge and Curcuma aromatica Salisb. inhibits liver metastasis of colon cancer by regulating EMT via the CXCL8/CXCR2 axis and PI3K/AKT/mTOR signaling pathway
Source: Chin Med. 2022 Aug 3;17:91. doi: 10.1186/s13020-022-00641-4 (PMC9351103; doi:10.1186/s13020-022-00641-4)
Supplement: Supplementary file 1 — Additional file 1: Additional tables and figures. Table S1. The MRM transitions and parameters of 13 compounds. Table S2. Regression equations and linear ranges of UPLC-QQQ-MS/MS. Figure S1. The total chromatograms of AC water extract and AC-containing serum with MRM mode. Figure S2. The chromatograms of AC water extract with the representative active ingredients. Figure S3. The chromatograms of AC-containing serum with the representative active ingredients. Table S3. Primer sequences involved in the experiment. [file 13020_2022_641_MOESM1_ESM.docx]

***Supplementary Material***

***Astragalus mongholicus* Bunge and *Curcuma aromatica* Salisb. inhibits liver metastasis of colon cancer by regulating EMT via the CXCL8/CXCR2 axis and PI3K/AKT/mTOR signaling pathway**

Fuyan Liu^1^, Yan Liang^1^, Ruolan Sun^1^, Weicheng Yang^2^, Zhongqing Liang^1^, Junfei Gu^1^, Fan Zhao^1^, Decai Tang^1^*

^1^ School of Traditional Chinese Medicine and School of Integrated Chinese and Western Medicine, Nanjing University of Chinese Medicine, Nanjing, China.

^2^ School of Chinese Materia Medica, Nanjing University of Chinese Medicine, Nanjing, China.

*** Correspondence:**
Decai Tang
talknow@njucm.edu.cn

# Quality control of AC water extract and AC-containing serum

## Sample and Standards Preparation

Preparation of water extract samples: 1 mL of AC water extract was mixed well by vortexing with 1 mL of methanol, and the supernatant was extracted and centrifuged at 12,000 rpm and 4℃ for 10 min to obtain the AMCW Water extract for the test. Before transferring to UPLC-MS analysis, dilute to 1mg/ml and filter through 0.22 μm membrane.

Preparation of AC serum sample: 100 µL of drug-containing serum sample was vortexed with 2mL of methanol for 120 s and centrifuged at 13,000 rpm and 4°C for 15 minutes. Subsequently, the supernatant with serum was collected and stored at -20°C.

Chemical standards of *Astragalus mongholicus* Bunge (1)Calycosin-7-glucoside(Y27F9H54731), (2)Isoferulic acid(BKR10798), (3)Ononin(R28O8F46957), (4)Calycosin(Y24N9Y75652), (5)Isomucronulatol 7-O-Glucoside(B21840), (6)Kaempferol(C26J8Y38642), (7)Formononetin(F27J7S18516), (8)Astragaloside Ⅳ(J04M8T30363), (9)Astragaloside I(C16J8G37958), (10)Astragaloside Ⅲ (B20567) were all provided by Yuanye Biotechnology Co. (Shanghai, China); For the standards of *Curcuma aromatica* Salisb., (11) Curcumin (LW17091410), (12) Demethoxycurcumin (LW16090803), (13) Bisdemethoxycurcumin (LW16090905) were purchased from Nanjing Liangwei Biotechnology Co., and the purity of each standard was over 98%. All working solutions were prepared by diluting the stock solutions with methanol.

## Chromatography and mass spectrometry conditions

The analyses were performed using a Waters ACQUITY UPLC instrument (Waters, Milford, MA, USA) equipped with a binary pump solvent delivery system, an autosampler and an online degasser. An ACQUITY UPLC BEH C18 column (100 mm × 2.1 mm, 1.7 μm) was applied for all the analysis. The mobile phase consisted of A (0.1% aqueous formic acid) and B (acetonitrile) using a gradient elution, which programmed as follows: 0∼3 min, 95 %∼55 % A; 3∼13 min, 55 %∼5% A; 13∼14 min, 5%A; 14∼14.1 min, 5%∼95 %A; 14.1∼15 min, 95 %A. The flow rate was set at 0.30 mL/min with 1 μL per injection. The column and autosampler temperature were conditioned at 40 °C and 4 °C, respectively.

Xevo Triple Quadrupole tandem quadrupole mass spectrometer (Waters, Milford, MA, USA) equipped with an ESI source was used to acquire the quantitation data. Parameters in the source were set as follows: capillary voltage 3.0 KV; source temperature 150 °C; temperature 400 °C; cone gas flow 50 L/h; desolvation gas flow 1000 L/h. Quantification was performed under multiple reaction monitoring (MRM) mode, and the data were acquired and processed using Waters Mass LynxTM Software v. 4.1.

## Identification and quantification

Identification of the target peaks was performed by comparing their UPLC retention times (RT) and m/z values with those of the reference compounds. To further confirm the structures of the constituents, standards and samples were analyzed by UPLC-QQQ-MS/MS. Quantification was performed using linear calibration plots of peak areas and concentration.

## Results

The MRM transitions and parameters of 13 compounds are shown in **Table S1**, and the typical chromatograms with MRM mode are presented in **Figure. S1**.

The standard solution was diluted to prepare a series of appropriate concentrations for the establishment of calibration curves. Calibration curves were constructed from peak areas versus their corresponding concentrations of the reference standards. The results are shown in **Table S2**. The r^2^ values were all above 0.990, indicating good linearity of the components to be tested.

The contents of (1) Calycosin-7-glucoside, (2) Isoferulic acid, (3) Ononin, (4) Calycosin, (5) Isomucronulatol 7-O-Glucoside, (6) Kaempferol, (7) Formononetin, (11) Curcumin, (12) Demethoxycurcumin, (13) Bisdemethoxycurcumin in the extract were 1.937, 0.897, 6.809, 6.067, 22.940, 0.421, 0.426, 0.366, 0.413, 0.294μg/ml, and the contents in the serum of rats were 0.143, 0.0912, 0.602, 0.106, 0.115, 0.040, 0.072, 0.061, 0.027, 0.056 μg/ml, respectively. (8) Astragaloside Ⅳ, (9) Astragaloside I, (10) Astragaloside Ⅲ were only detected in AC extracts at the levels of 47.263, 111.194, 80.816 μg/ml respectively.

**Table S1. The MRM transitions and parameters of 13 compounds**

| Compound | Polarity | Precursor （m/z） | Product （m/z） | Collision Energy （V） | Retention time（min） |
| --- | --- | --- | --- | --- | --- |
| 1. Calycosin-7-glucoside | ESI+ | 447.1 | 270.02 | 20 | 3.29 |
| 1. Isoferulic acid | ESI- | 193 | 134 | 14.47 | 3.38 |
| 1. Ononin | ESI+ | 453.1 | 290.929 | 19.06 | 4.65 |
| 1. Calycosin | ESI- | 283.05 | 267.97 | 18.53 | 5.05 |
| 1. Isomucronulatol 7-O-Glucoside | ESI- | 463.1 | 301.054 | 16.37 | 5.18 |
| 1. Kaempferol | ESI- | 285.05 | 238.917 | 27.17 | 5.72 |
| 1. Formononetin | ESI+ | 267.038 | 251.97 | 20.39 | 6.22 |
| 1. Astragaloside Ⅳ | ESI+ | 783.3 | 628.25 | 48.82 | 6.43 |
| 1. Astragaloside I | ESI+ | 892.35 | 712.25 | 48.56 | 7.36 |
| 1. Astragaloside Ⅲ | ESI- | 783.2 | 489.31 | 33.66 | 6.43 |
| 1. Curcumin | ESI- | 367.1 | 217.018 | 10.23 | 7.43 |
| 1. Demethoxycurcumin | ESI- | 337.05 | 217.042 | 10.23 | 7.32 |
| 1. Bisdemethoxycurcumin | ESI- | 307.05 | 186.917 | 10.227 | 7.22 |

**Table S2. Regression equations and linear ranges of UPLC-QQQ-MS/MS.**

| **NO.** | **Standards** | | **Calibration curves** | **r^2^** | **Linear range (ng/mL)** | **Component content (ng/mL)** | | |
| --- | --- | --- | --- | --- | --- | --- | --- | --- |
|  |  |  |  |  |  | **Water extract** | **AC-containing serum** | |
| 1 | Calycosin-7-glucoside | y = 162.17x - 1040.8 | | 0.9993 | 1-1000 | 1936.733 | | 142.665 |
| 2 | Isoferulic acid | y = 649.18x - 2718.3 | | 0.9979 | 1-1000 | 897.024 | | 91.232 |
| 3 | Ononin | y = 22.102x - 115.82 | | 0.9931 | 5-500 | 6808.524 | | 602.498 |
| 4 | Calycosin | y = 11173x - 22803 | | 0.9941 | 1-100 | 6067.439 | | 106.013 |
| 5 | Isomucronulatol 7-O-Glucoside | y = 1143.2x - 5796.8 | | 0.9990 | 5-500 | 22939.801 | | 114.937 |
| 6 | Kaempferol | y = 125.27x - 108.08 | | 0.9977 | 5-500 | 421.330 | | 39.607 |
| 7 | Formononetin | y = 25719x - 43807 | | 0.9983 | 5-100 | 425.833 | | 71.865 |
| 8 | Astragaloside Ⅳ | y = 29.4502x + 105.55 | | 0.9982 | 5-500 | 47263.021 | | _ |
| 9 | Astragaloside I | y = 3.0173x + 53.955 | | 0.9964 | 25-1000 | 111193.617 | | _ |
| 10 | Astragaloside Ⅲ | y = 113.01x - 459.59 | | 0.9986 | 5-500 | 80816.37908 | | _ |
| 11 | Curcumin | y = 5915.6x - 16842 | | 0.9932 | 5-100 | 365.998 | | 61.387 |
| 12 | Demethoxycurcumin | y = 7383.7x - 9699.3 | | 0.9943 | 5-100 | 413.239 | | 27.150 |
| 13 | Bisdemethoxycurcumin | y = 918.42x - 2574.9 | | 0.9913 | 5-100 | 293.863 | | 56.268 |


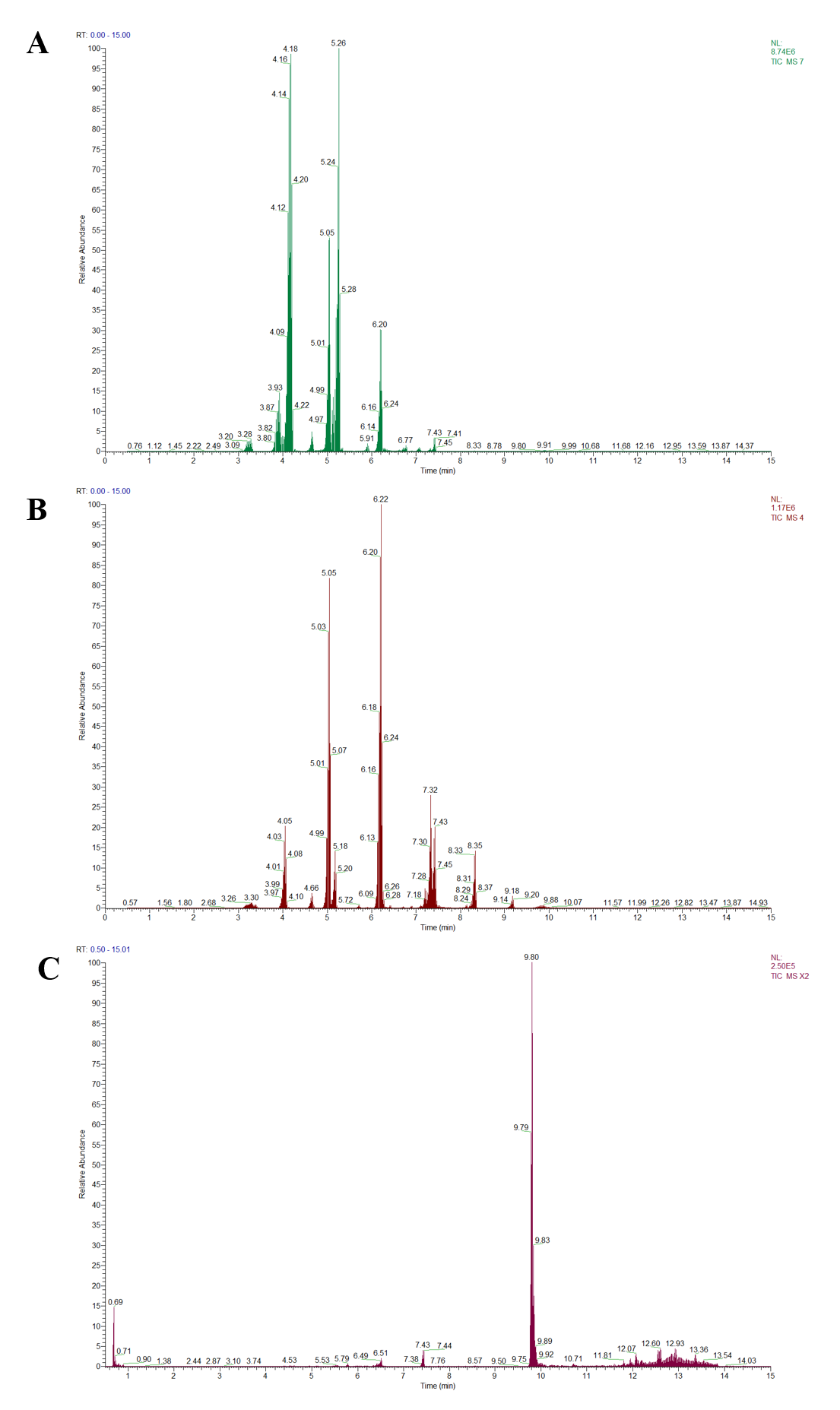


**Figure S1. The total chromatograms of AC water extract and AC-containing serum with MRM mode.** (A. green, AC water extract; B. red, standards; C. purple, AC-containing serum)

**Figure S2**


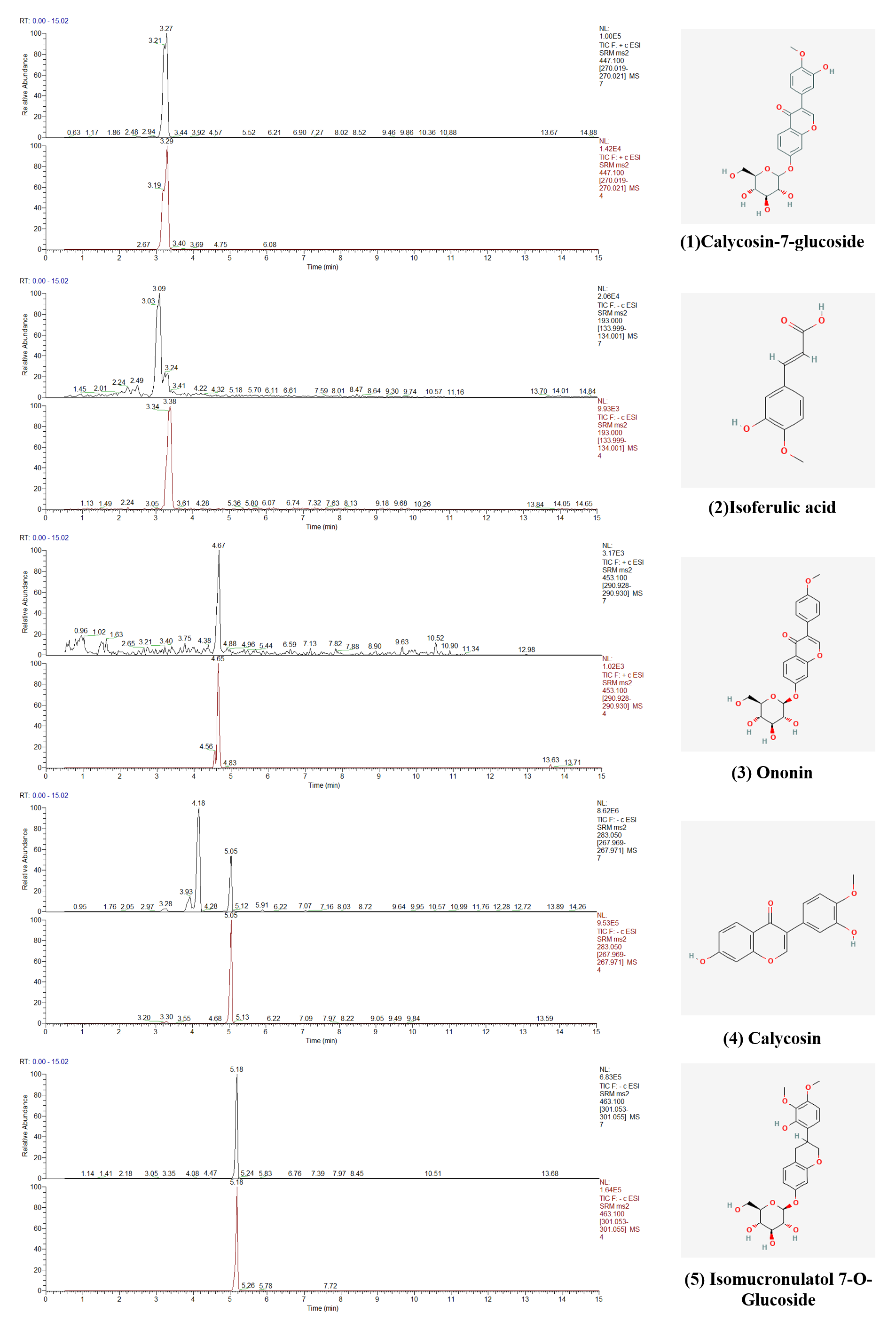


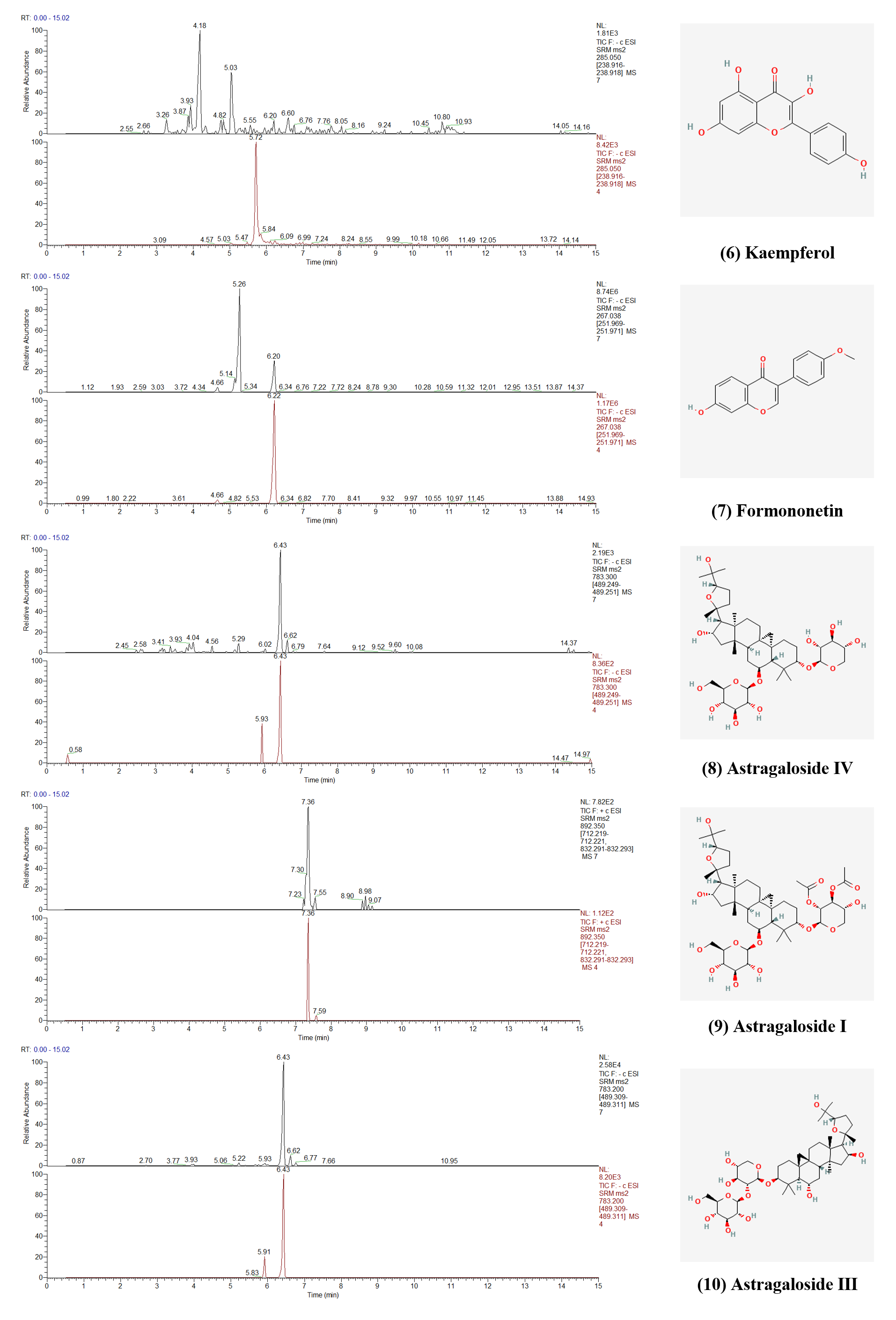


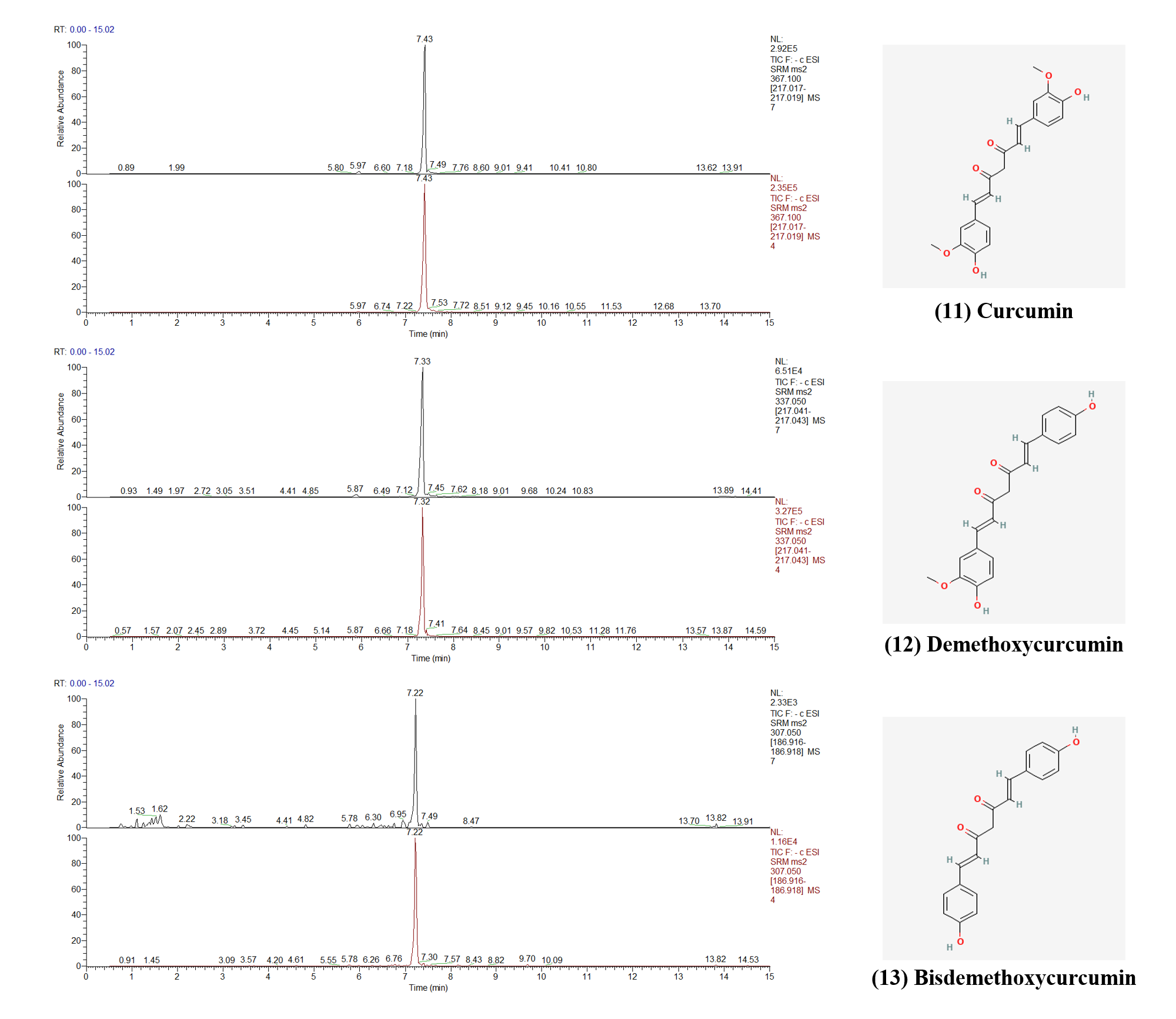


**Figure S2. The chromatograms of AC water extract with the representative active ingredients.** (black, AC water extract; red, standards)

**Figure S3**


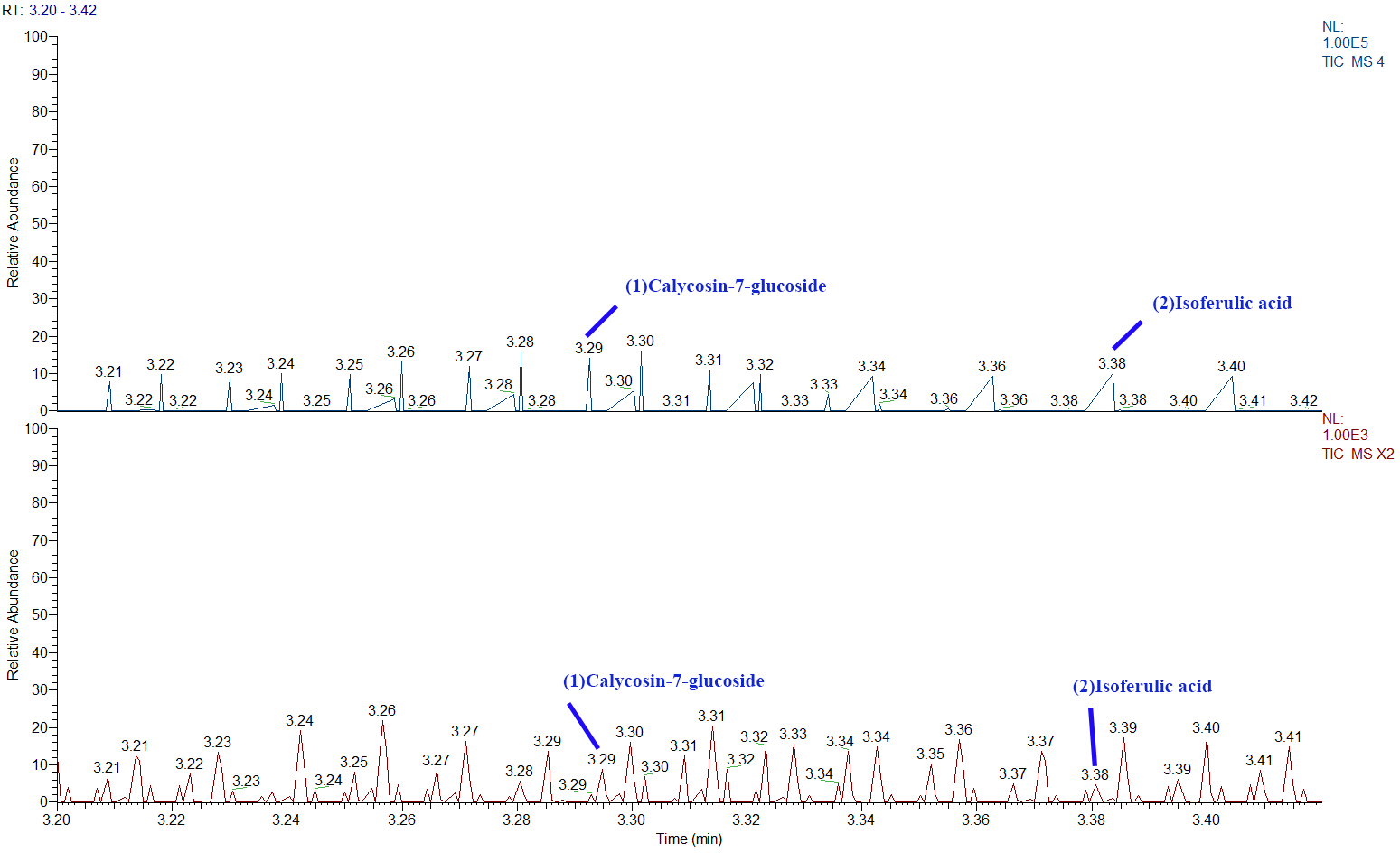


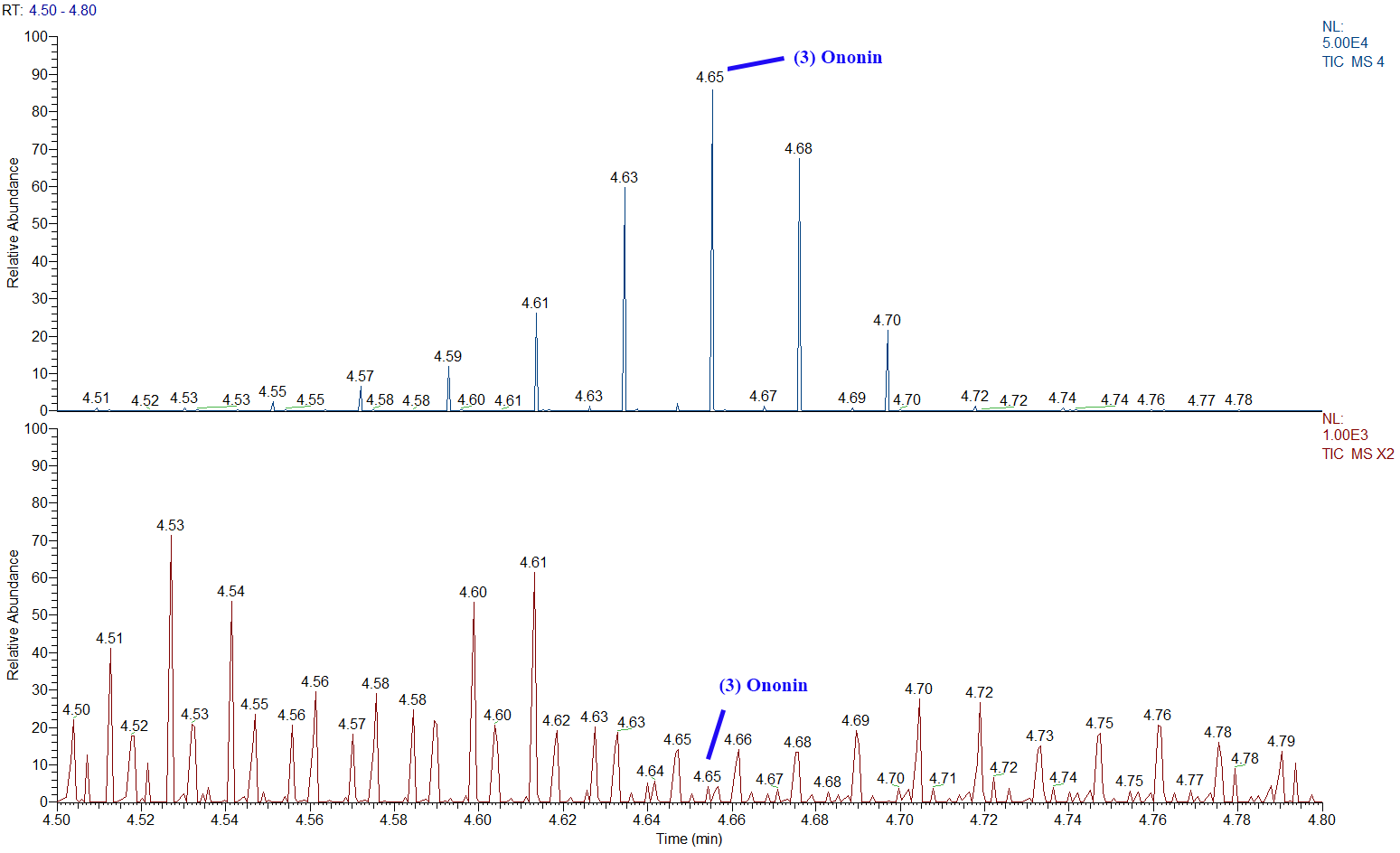


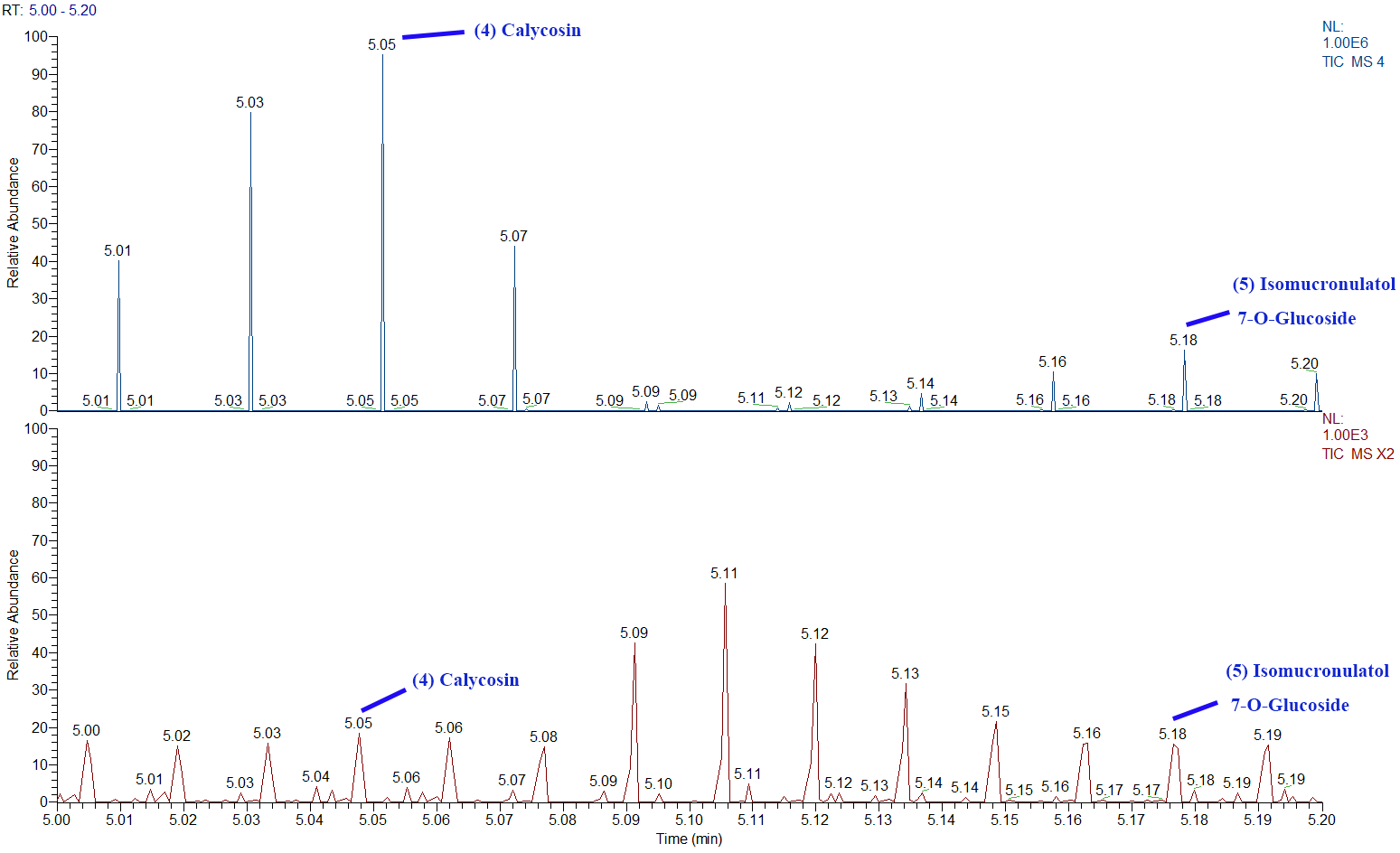


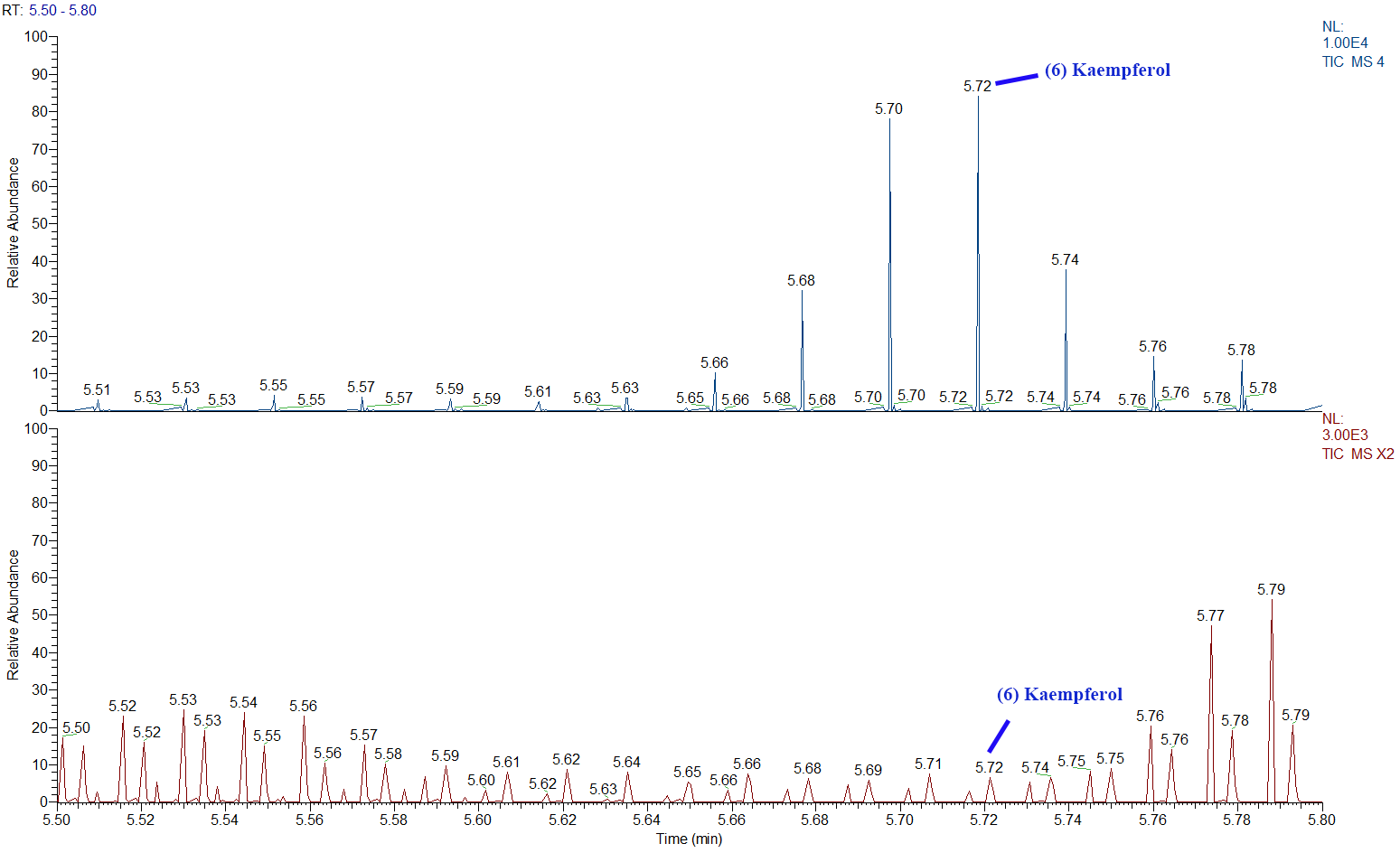


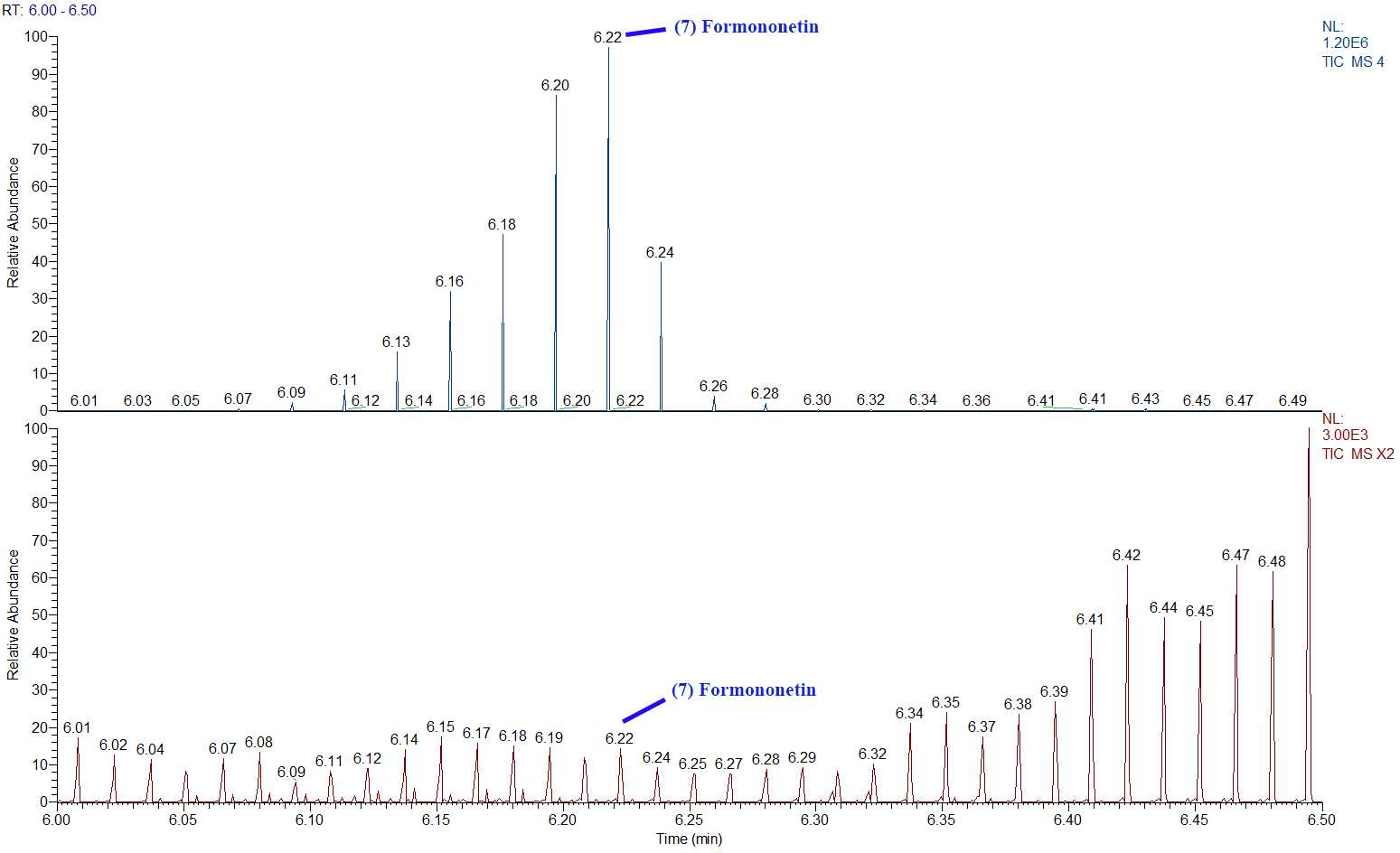


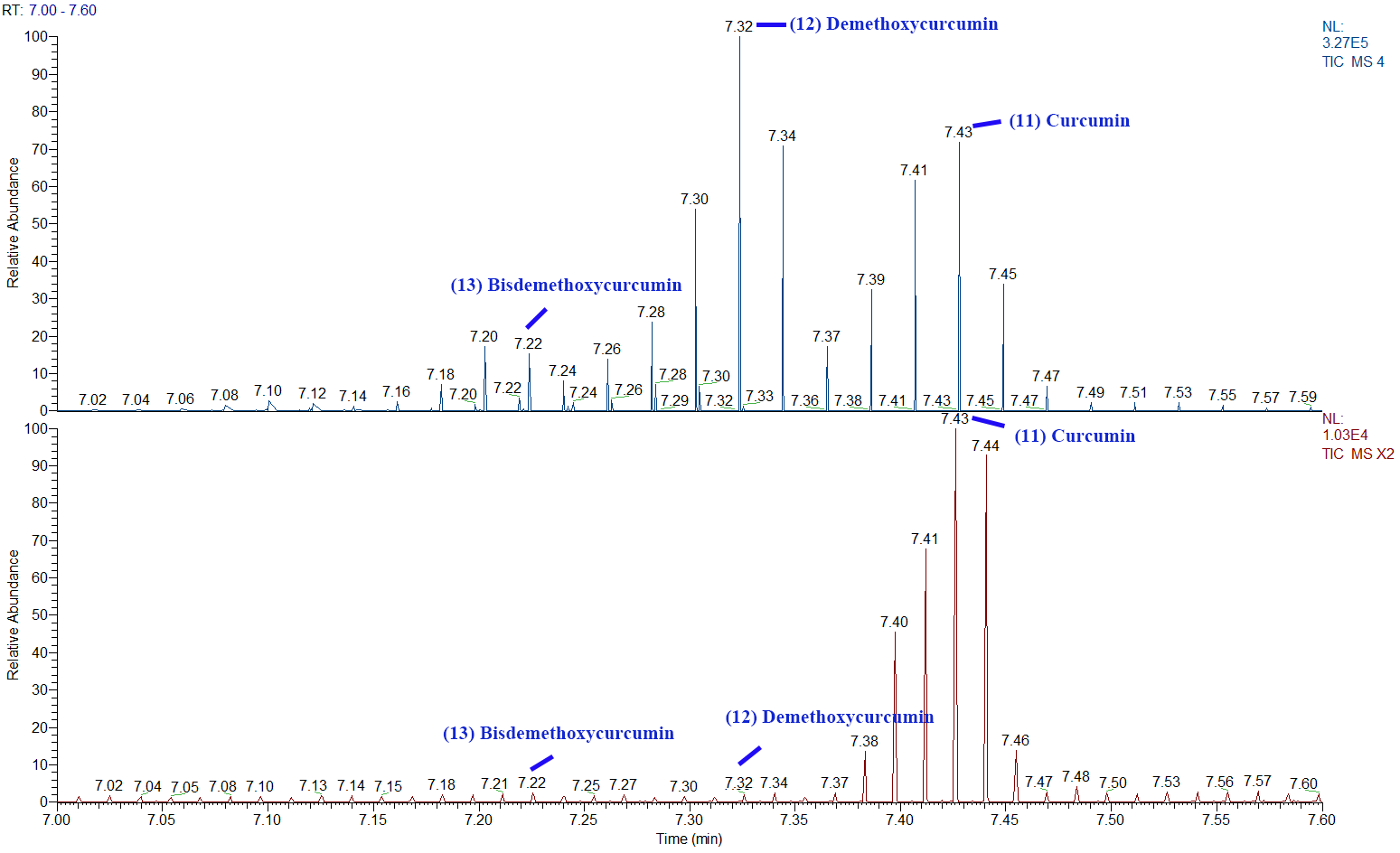


**Figure S3. The chromatograms of AC-containing serum with the representative active ingredients.** (blue, standards; red, AC-containing serum)

# Supplementary Table S3. Primer sequences involved in the experiment

| **Gene** | **Primer_sequence (5'-3')** | **Organism** | **Base_number** |
| --- | --- | --- | --- |
| CXCL8 | F: AGGTCGGTGTGAACGGATTTG  R: TGTAGACCATGTAGTTGAGGTCA | Mouse | 21  23 |
| CXCR1 | F: TCTGGACTAATCCTGAGGGTG  R: GCCTGTTGGTTATTGGAACTCTC | Mouse | 21  23 |
| CXCR2 | F: TCGAGACCATTTACTGCAACAG  R: CATTGCCGGTGGAAATTCCTT | Mouse | 22  21 |
| PI3K | F: ACACCACGGTTTGGACTATGG  R: GGCTACAGTAGTGGGCTTGG | Mouse | 21  20 |
| AKT | F: ATGAACGACGTAGCCATTGTG  R: TTGTAGCCAATAAAGGTGCCAT | Mouse | 21  22 |
| mTOR | F: CAGTTCGCCAGTGGACTGAAG  R: GCTGGTCATAGAAGCGAGTAGAC | Mouse | 21  23 |
| E-cadherin | F: CAGTTCCGAGGTCTACACCTT  R: TGAATCGGGAGTCTTCCGAAAA | Mouse | 21  22 |
| N-cadherin | F: AGGCTTCTGGTGAAATTGCAT  R: GTCCACCTTGAAATCTGCTGG | Mouse | 21  21 |
| vimentin | F: CGTCCACACGCACCTACAG  R: GGGGGATGAGGAATAGAGGCT | Mouse | 19  21 |
| snail | F: CACACGCTGCCTTGTGTCT  R: GGTCAGCAAAAGCACGGTT | Mouse | 19  19 |
